# The ANEMONE: Theoretical Foundations for UX Evaluation of Action and Intention Recognition in Human-Robot Interaction

**DOI:** 10.3390/s20154284

**Published:** 2020-07-31

**Authors:** Jessica Lindblom, Beatrice Alenljung

**Affiliations:** School of Informatics, University of Skövde, Box 408, 541 28 Skövde, Sweden; beatrice.alenljung@his.se

**Keywords:** human-robot interaction, human-robot collaboration, user-centered, evaluation, action recognition, intention recognition, activity theory, seven stages of action, user experience (UX)

## Abstract

The coexistence of robots and humans in shared physical and social spaces is expected to increase. A key enabler of high-quality interaction is a mutual understanding of each other’s actions and intentions. In this paper, we motivate and present a systematic user experience (UX) evaluation framework of action and intention recognition between humans and robots from a UX perspective, because there is an identified lack of this kind of evaluation methodology. The evaluation framework is packaged into a methodological approach called ANEMONE (action and intention recognition in human robot interaction). ANEMONE has its foundation in cultural-historical activity theory (AT) as the theoretical lens, the seven stages of action model, and user experience (UX) evaluation methodology, which together are useful in motivating and framing the work presented in this paper. The proposed methodological approach of ANEMONE provides guidance on how to measure, assess, and evaluate the mutual recognition of actions and intentions between humans and robots for investigators of UX evaluation. The paper ends with a discussion, addresses future work, and some concluding remarks.

## 1. Introduction

Several kinds of robots are entering the world we live in and they are expected to be fully integrated in future society. As recently foreseen by Stephanidis et al. [1], advanced forms of technology will be omnipresent, where robots “*predict* and *anticipate* human needs, robotic systems are an integral part of everyday life, and *humans’ abilities are technologically supported*” [1] (p. 1229). These robots will be situated and embedded in technologically enriched environments, in which information will be exchanged ‘*naturally*’ between humans and robots, resulting in hybrid worlds where humans coexist in the digital and the real world [1]. In order to become a natural part in the daily life of humans in domestic, vocational, as well as public contexts, interaction and coexistence with several kinds of robots has to be experienced positively by humans while also being suited to our purposes, with the interaction being smooth and satisfying [2,3,4,5]. This means that humans should experience that a robot delivers according to existing explicit and implicit goals, which means that it performs efficiently, and in a way that makes humans feel trust, safety, and convenience while being together [5].

Similarly, Stephanidis et al. [1] argue that this development leads to new challenges for human-centered approaches, which “call for shifts in both focus and methods, in order to formulate and address the critical issues that underlie a more trustful and beneficial relationship between humankind and technology” [1] (p. 1230). This shift in focus on *methods* as well as the focus on *action* and *intention recognition* between humans and robots is the major topic of this paper. We present a systematic evaluation framework of action and intention recognition between humans and robots from a user experience (UX) perspective, because we have identified a lack of this kind of evaluation methodology. We denote this methodological approach ANEMONE, which is the acronym for **A**ction a**N**d int**E**ntion Recognition in hu**M**an r**O**bot i**N**t**E**raction. The issue of mutual action and intention recognition between humans and robots as well as the motivations for the ANEMONE approach is elaborated on in the upcoming two sub-sections.

### 1.1. The Role and Relevance of Mutual Action and Intention Recognition between Humans and Robots in Human-Robot Interaction (HRI)

The main purpose of autonomous systems like robots is to, e.g., enable humans to perform tasks they could not do in proper ways earlier and facilitate monotonous or risky tasks, as well as offering assistance, companionship, or enjoyment [6]. Hence, various kinds of robots provide several forms of added value, which could range from doing repetitive tasks in manufacturing assembly, vacuuming the flat, assisting as companions to older people to serving as educational robots for preschoolers. In some of those cases, the human users seldom have to constantly interact with the robot, but other robot usage situations, like assisting older people or collaborating in assembly tasks on the shop floor, require more recurrent, continuous, and multi-faceted interaction between humans and robots. This means that these more complex interplays between robots and their intended users have to be carefully considered when designing and developing robots in order for them to provide the added value to intended human users, depending on the situation, task at hand, and the context of use [6]. The challenge of understanding and designing a natural, fluent, and trustworthy interaction between human(s) and robot(s) is the key factor of interest in the field of human-robot interaction (HRI) [6,7,8,9,10]. More specifically, Dautenhahn [10] characterizes HRI as follows:

“HRI is the science of studying people’s behavior and attitudes towards robots in relationship to the physical, technological and interactive features of the robots, with the goal to develop robots that facilitate the emergence of human-robot interactions that are at the same time efficient (according to original requirements of their envisaged area of use), but are also acceptable to people, and meet the social and emotional needs of their individual users as well as respecting human values” [10].

According to Fong et al. [11], socially interactive robots are briefly characterized as “robots for which social interaction plays a key role” while interacting with humans (p. 145). During the last two decades, research in the cognitive sciences fields, in particular embodied social cognition and cognitive neuroscience, has witnessed profound advancement in elucidating the underlying mechanisms of the recognition of actions and intentions in social interactions between humans that is fundamental to mutual interaction between humans, in which the mirror neuron system plays a significant role (e.g., [12,13,14,15,16,17,18,19,20,21,22,23,24,25,26,27,28]). As pointed out by Vernon, Thill, and Ziemke [26], among others, there is a huge challenge to accomplish analogous mutual as well as fluent action and intention recognition between humans and robots as in social human-human interaction [17,18,19,20,21,22,23,24,25,26,27,28], due to the apparent differences between the fundamental biological mechanisms in living human beings compared to the technological ones currently used in robots [17,19,25,26,27,28,29]. From a more embodied social cognition perspective, there is a major difference in how living agents enact a social world based on the underlying sensori-motor processes, particularly the mechanisms of the mirror neuron system, compared to the electrical wirings and technological implementation of artificial cognitive agents like robots [25,26,27,28,29]. Some recent research in HRI has investigated and analyzed whether, and to what extent, a similar smooth and fluent understanding as in social human-human interaction could also arise in social human-robot interaction, and there is some evidence where robots have been considered and experienced as goal-oriented agents by human participants [30,31,32,33,34].

It is argued that mutual interaction between humans and humans is needed if robots should be considered as partners instead of as tools [17,19,30,35,36,37], but to what extent they need to grasp the intentions of others is a much debated issue. However, at least it is argued that to achieve some kind of action and intention recognition between humans and robots, which possibly is a pre-requisite for some basic social interaction skills [24,26,28,30], is necessary for developing into engaging in more advanced forms of social interaction such as joint actions and mutual collaboration [38,39,40,41,42,43,44,45,46,47,48,49,50,51,52,53,54,55]. In other words, it requires that robots are able to perceive similar emotional and behavioral patterns and environmental cues as humans do (e.g., [1,5,53,54,55]). Accordingly, it has been acknowledged that the *interaction quality* between humans and robots has to progress to a sufficient degree that it is comparable to the fluent, trustworthy, and smooth interaction currently accomplished between humans [52], which implies that the robot has to act autonomously to some extent. Although autonomous action is a core aspect for many kinds of robots, the autonomy concept is considered rather problematic, because it has received substantially separate interpretations within different research communities [53,54,55]. In the traditional industrial robotics community, on the one hand, high autonomy suggests that the robot’s behavior can be stipulated by the human operator. In biology, the embodied cognitive science, and to some extent also in HRI communities, on the other hand, the autonomy concept refers to an autonomous robot that is being viewed as an artificial cognitive agent where its actual behavior cannot be completely regulated by the human operator [53,54,55].

In the fields of HRI and robotics, there has been a lot of research on robots identifying, understanding, and predicting human intention and actions (e.g., [1,17,18,19,20,24,33,39,40,41,45,50,51,52]). It should be noted, however, that there is little existent work where robots are able to fully satisfy the requirements for having recognition capacities, although they display some aspect of recognition. It still is not an intrinsic capacity of the robot system itself, rather the recognition capacity has been added in specific situations [52], designed from the outside by the human designer [53,54,55,56]. As pointed out by Mindell [56], the robot or another kind of artificial intelligent system, is still wrapped by human control. Mindell [56] points out that it is still the robot designer’s beliefs, ideas, and underlying intentions that are designed and implemented into the autonomous robot system, which means that every human user who is interacting with the robot in practice is interacting with the designer who is still ‘being there’ in the robot, although implicitly present. Consequently, this means that the way a particular robot actually behaves, although not always anticipated beforehand, is within the robot designer’s inflicted constraints. As phrased by Mindell [56] (p. 10): “How a system [robot] is designed, by whom, and for what purpose shapes its abilities and its relationships with the people who use it”. A robot might say hello to a human since the greeting is part of its behavioral script but not an intrinsic part of it in trying to establish mutual recognition as being key in social interaction and joint action between human beings [51,52,53,54,55,56].

According to Brinck and Balkenius [52], there are three steps to achieve mutual recognition. The first and most important step is identification, in which the other individual is identified based on directly perceptible attributes, e.g., movement or action, gaze, verbal utterance, gesture, and emotion, or other properties. This information could be used to form expectations “here and now” of the forthcoming interaction, and it could also be used to infer the short-term or long-term goal(s) of the other individual [52]. Identification does not require that every single attribute of the other is needed to be correctly identified. It could also be based on previous interactions as well as being context-dependent and shaped by the kind of interaction that unfolds. The second step is confirmation, in which the other confirms that identification has occurred, and it could be manifested by subtle or more explicit signals, showing that the attention directed towards the other individual is established and simultaneously being attended to [52]. Mutual recognition is achieved when the interactive individuals’ behavior could be influenced by the other as well as when there is a willingness to be influenced. Achieving mutual recognition is a crucial step towards establishing a sense of understanding of the other’s intentions. The third and final step is turn-taking, in which a simultaneous, immediate, and dynamic coupling of human and robot occurs that unfolds smoothly, where the behavior and actions of each individual is dependent on those of the other individual [25,52,55,57].

However, as robots are entering the fuzzy world of people, where the coexistence and turn-taking goes beyond structured interaction between one human and one robot in a controlled area [13], it is also essential that that humans can read robots in terms of what their intentions and actions are and mean [14,15,16]. The concept of intentions has been widely disputed, i.e., could an artifact, such as a robot, have intentions or not (e.g., [4,15,17,18,20,25,28]). The intentional system theory by Dennett [58] provides a pragmatic way for HRI to deal with this matter. Dennett [58] argues that “Anything that is usefully and voluminously predictable from the intentional stance is, by definition, an intentional system. The intentional stance is the strategy of interpreting the behavior of an entity (person, animal, artifact, whatever) by treating it as if it were a rational agent who governed its ‘choice’ of ‘action’ by a ‘consideration’ of its ‘beliefs’ and ‘desires’” [58] (p. 339). Thus, what matters is that the human users take an intentional stance towards robots, not whether or not robots have intentions. This stresses the necessity for, e.g., the well-designed appearance, functionality, and behavior of a robot in relation to its context in order to make it possible for a human to smoothly perceive, grasp, comprehend, and predict its intentions and actions. This will make it more likely that the person will positively experience the robot, since, for instance, the sense of control can be expected to increase [59]. A central part in order to succeed in the task of using the intentional stance while interacting with robots is the demand on human users to easily and effortlessly perceive, experience, understand, and predict the robot’s intentions and actions [51,52]. As pointed out by Brinck and Balkenius [52], among others, we therefore argue that the mutual recognition of actions and intentions between humans and robots is important for high-quality coexistence in society.

However, the envisioned smooth and natural mutual recognition of actions and intentions between humans and robots does not materialize automatically. Therefore, it is of major importance when designing and developing robots to systematically evaluate if humans can properly recognize the actions and intentions of robots in order to identify problems of robot usage in various kinds of human environments, e.g., offices, factories, hospitals, museums, and educational settings. The foreseen and increased level of robot participation in everyday human activities obviously emphasizes the role and relevance of systematically evaluating the *interaction quality* between the human user(s) and the robot(s) from a UX perspective [5,60,61,62,63,64,65,66,67]. Indeed, the prominent HRI scholar Dautenhahn [68] highlights that currently her greatest concern of empirical HRI research is that the field of experimental psychology appears repeatedly to be viewed as the ‘one and only’ and, accordingly, the golden standard method for performing proper HRI work. Dautenhahn [68] stresses that besides the obvious pros of experimental HRI research, this way of doing research rarely provides answers on how human users in real-world environments would interact with a robot in practice. Dautenhahn [68] points out that obtained results from HRI studies should also rely on real-world circumstances and also on the specific tasks human users are carrying out combined with the complexity of interacting with an, every now and then unpredictable, robot that juggles between several tasks simultaneously [68]. Dautenhahn [68] admits that the above suggested HRI research approach does not fit well with the prevailing method paradigm of HRI studies greatly influenced by experimental psychology. Moreover, experimental HRI studies do not provide any explicit suggestions of how to overcome any identified problems in the interaction between humans and robots, and seldom consider the nature of the task(s) carried out, or to what extent the context of usage matters [64]. Dautenhahn [68] declares that she personally would like to see more empirical HRI studies that involve more authentic and less trivial interactions between humans and robots as well as encompassing more complex robot behaviors, which are carried out and situated within ecologically valid environments. We totally agree with Dautenhahn’s opinion [68] that these kind of empirical HRI studies are not easy to conduct in practice, but there is still a necessity for the field of HRI to complement and expand beyond the experimental psychology research lab in order to have implications for societal relevance and social impact in society.

Hence, we suggest taking a UX view that comprises a systematic evaluation of the *interaction quality* between human(s) and robot(s) is of major concern in order for autonomous systems like various kinds of robots to offer long-term added values to people’s professional and private lifes. We argue that it is of major importance that socially interactive robots, in similar ways as any digitally interactive devices, services, and/or systems, achieve a positive or great user experience in order to accomplish the intended benefits and foreseen societal relevance of HRI in various usage settings [5,60,61,62,63,64,65,66,67]. Generally speaking, UX refers to the feelings that occur and unfold inside the human users during the usage of interactive technology in a particular context of use [69,70,71,72]. If the usage of socially interactive robots causes a negative or bad user experience for the intended end-users in the context of use, it could result in several undesirable negative consequences, e.g., unwillingness to use the actual robot, incorrect handling, serious safety issues, perceived mistrust, or spreading of bad reputation of socially interactive robots in general [60,61,62,63,64,65,66,67,68].

### 1.2. Motivations and Aim for ANEMONE

Some evaluation frameworks that consider an overall human-centered perspective, i.e., focus on the intended end-users of the robot, in the interaction between humans and robots already exist (e.g., [73,74,75]). Recently, Hoffman [73] focuses on fluency as an indicator of the quality of the interaction in human-robot collaboration (HRC). He states that fluency, although being a rather vague concept, occurs when humans and robots collaborate on a shared task, in which they are familiar with the activity and each other and are able to acquire a high level of coordination, resulting in precise and efficient timing, and adapt to and alter their plans and actions continuously while collaborating on a shared activity [73]. However, Hoffman emphasizes that future research needs to investigate how one should consider and handle correct and incorrect actions of the robot and the human and identify the causes behind these mismatches that hinder fluency in interaction. These mismatches could be interpreted to be aligned with action and intention recognition mechanisms between humans and robots, which we argued in Section 1.1. These mechanisms may be the underlying pre-requisite for achieving mutual and subsequently ‘fluent’ interaction between humans and robots. Garvasi et al. [74] have developed a general conceptual framework to evaluate the collaboration between humans and robots that encompasses many aspects of collaboration, in which human-centered usability issues are addressed. Both Hoffman [73] and Garvasi et al. [74] focus on some human-centered aspects of the human operator in these present developed evaluation frameworks, but we intend to extend their human-centered approaches to a UX approach, based on the following motivations.

It should be pointed out that the human-centered approach, as characterized in the International Organization for Standardization’s (ISO) specification for the ergonomics of human system interaction—part 210: human-centered design for interactive systems [70] includes both the usability and the UX perspectives. This means that the fields of human factors, human-computer interaction (HCI), and UX share some common similarities, i.e., focusing on the end-users, but there are also some differences, based on underlying theoretical perspectives on how to conceptualize the human users in human-technology interaction [76,77,78,79,80]. Bannon [77] already in the early 1990s created the well-known catchphrase “from human factors to human actors”, in which he highlights the conceptual shift in considering how to view the human users in human-technology interaction in general. Bannon’s take-home message is that the human users were commonly viewed as human factors, i.e., more passive elements or cogs in the human-technology interaction loop. Instead, he advocates the then emerging view that considers human users as active human actors, having their own agendas, motives, and goals while interacting with technology [77,78]. This proposed action perspective is well-aligned with the more modern approaches of human cognition, which stress that cognition is for action and anticipation of action instead of more passive information-processing (e.g., [12,13,14,15,16,25,28,29,30,53,76]). Indeed, it should be acknowledged that human factors is a dominant and well justified research field that has been successful for a long time, particularly in safety-critical systems, but where the common practice is to mainly focus on usability in the form of performance-related aspects although well-being is an integrated part of human factors [79,80,81]. Therefore, it has been argued that one current challenge of human factors is that that they are not well-aligned with the more modern approaches for studying human cognition and technology-mediated interaction, such as the cultural-historical activity theory (AT) [82,83,84,85,86], embodied cognition [29,81], and UX [79,80,81]. The fields of human factors and HCI have evolved as the technologies have become more advanced and additional ways of using technology have emerged both at work and in private life [1,5,69,71,72,76,82,83,84,85,86]. This development has resulted in several waves of HCI research, where UX and the more modern understandings of cognition are considered as the third wave and beyond [79,80,81]. It should be emphasized that we do not intend to start an argument about the best way of doing research or evaluation in HRI, but rather wish to emphasize that different approaches and disciplines could shed complementary perspectives on several aspects of the phenomena of doing evaluations in HRI. Grundgeiger et al. [81] argue that considering and improving users’ experience (UX) is one of the fundamental aims of ISO standards on human factors and human-computer interaction (HCI) [70]. A prominent and well-developed evaluation approach for HRI is the USUS (abbreviation of Usability, Social acceptance, User experience, Societal impact) framework [75,87], which includes UX aspects that have been used in some evaluation studies [75,87]. However, USUS is very general in its approach and, apart from UX, it also embraces aspects such as societal impacts, e.g., working conditions and employment, and social acceptance, e.g., forms of grouping [75,87]. Although the USUS framework for evaluating HRI includes some aspects of user experience, we consider it too general, since it does not provide any theoretical foundation. The USUS framework also lacks UX goals, which are of major importance for a proper UX evaluation [61,63,64,65,71].

Thus, current evaluation methods for human-robot interaction and collaboration do no focus on action and intention recognition in HRI from a UX perspective, and we have identified a need for such an approach based on the following motivations. Firstly, there is a need to systematically *evaluate the quality* of the interaction from a UX perspective beyond human factors. Therefore, we will apply a UX perspective. Grundgeiger et al. [81], among others, provide several reasons why users’ experience should be considered when interacting with technology in general. They emphasize that interaction *per se* is an ongoing experience, implying that interaction with technology always has an associated UX, which is ubiquitously present whether or not it is explicitly addressed by researchers or designers [81]. Moreover, it is not enough to ensure pragmatic qualities, e.g., error reduction, effective performance, and reduced cognitive load, when humans are interacting with technology. Much more emphasis needs to be focused on hedonic qualities, e.g., satisfying humans’ motives and psychological needs, including expectations, emotions, and well-being [1,5,60,61,62,63,64,65,66,67,69,71,72,77,78].

Secondly, we try to narrow the existing gap in UX as well as in HRI research, which is the lack of theoretical frameworks in understanding user experience and human-robot interaction [88,89,90]. Grundgeiger et al. [81] point out that AT is a feasible approach for consider interaction that emphasizes a positive UX. By using AT, we also put the interaction between humans and robots within a context. Grundgeiger et al. [81] highlight that good interaction and UX is indicated by a useful and transparent tool, in our case the robot, which amplifies human capabilities, and by adopting AT [82,83,84,85,86], we are provided with both contextual information that is essential for gaining a deeper understanding of the contradictions and breakdowns in the interaction between humans and robots as well as considering the fulfillment of human’s needs and motives when interacting with robots.

Thirdly, neither current UX methods nor evaluation methods of HRI and HRC focus on or capture action intention recognition between humans and robots, which is fundamental for a smooth and fluent interaction to occur, as well as for identifying mismatches or breakdowns in the interaction. As mentioned above, both Stephanidis et al. [1] and Hoffman [73] have raised the need for such a framework to evaluate the mutual action and intent recognition between humans and robots.

In this paper, we suggest a path forward in terms of addressing the above identified theoretical and methodological challenges and needs in order to successfully evaluate the action and intent recognition of human-robot interaction and collaboration in a systemic way from a more modern UX perspective. The overall purpose of this paper is to provide inspiration and support to investigators that are interested in evaluating action and intention recognition between humans and robots from the UX viewpoint, with the starting point of less complex situations that could be expanded upon. Thus, the overall aim of this paper is to provide a theoretical foundation for UX evaluation of action and intention recognition in HRI, which is packaged into a methodological approach called ANEMONE. ANEMONE has its foundation in AT [81,82,83,84,85,86] as its theoretical lens, the seven stages of action model [91], and UX evaluation methodology (for an overview see, e.g., [5,71,72,92,93]), which together are useful in motivating and framing the work presented in this paper. The proposed methodological approach, ANEMONE, provides guidance on how to measure, assess, and evaluate the mutual recognition of actions and intentions between humans and robots for investigators of UX evaluation.

In particular, ANEMONE focuses on how to identify the possibilities for humans to perceive, understand, and predict the intentions and actions of a robot, as well as facilitates comprehension of why something works or not in the interaction. The intended users of the proposed methodological approach ANEMONE also include investigators with limited expertise in areas of cognitive ergonomics, human factors, cognitive science, HCI, or UX, as well as limited familiarity of UX evaluation, although we recommended some prior knowledge or experience in doing HCI usability or UX evaluations.

The remainder of this paper is structured as follows. The next section provides some clarifications regarding the evaluation method development process used for the design and development of ANEMONE, which is inspired by Blandford and Greene’s [94] work, which here functions as a kind of meta-methodology. We intend to be transparent about how the methodological approach of ANEMONE emerged, and therefore, we devote a section for this process. We return to their approach in the discussion section. We then provide a foundation in Section 3 that presents in more detail the building blocks of ANEMONE, having a foundation in AT as its lens, the seven stages of action model, and UX evaluation methodology, which together are useful in motivating and framing the ANEMONE approach presented in this paper. Next, in Section 4 we present the outcome of the underlying theoretical and methodological foundation for the methodological approach with a description of the methodological approach of ANEMONE and its procedure. The final section, Section 5, summarizes the lessons learned, discusses the contributions and limitations of the work carried out and the current version of ANEMONE, and also briefly addresses some future work.

## 2. Method Development Approach for ANEMONE

ANEMONE has emerged from an identified need—in our case, the lack of a methodological approach specifically focusing on how to evaluate the mutual recognition of actions and intentions between humans and robots from a UX perspective in HRI.

It should be highlighted that the concepts *method* and *methodology* are commonly used in a variety of ways, and this sometimes ends up in extensive inconsistences when the use of these concepts is intermixed. In order to clarify this issue, Blandford and Green consider [94] “a methodology as a collection of methods used in a systematic way, but of course at a higher level of abstraction that a collection of methods can be regarded as a ‘bigger’ method” (p. 161) (for further details on this topic, see [95]). ANEMONE is referred to as a methodology and not a single method in this paper. In order to further sort out the usage of the method phenomena, and how these concepts are used in a variety of ways in theory and practice, we are influenced by Lings’s and Lundell’s [96] recommendation of terminology [95]. They have coined the terms of ‘method-in-concept’, ‘method-in-tool’, and ‘method-in-action’, which reveal several interdepended perspectives on the method phenomenon. Broadly speaking, their term method-in-concept refers to the main underlying idea of the envisioned method/methodology as understood by its creators and takes into consideration the shared amount of values, ideas, and assumptions encapsulated in the method/methodology. Next, their term method-in-tool represents the fulfillment of the method-in-concept in the form of the description, instructions, procedure, handbook, and (digital) tools used to accomplish the conceptualized idea of the method/methodology. Method-in-action represents the practical usage of the method(s)/methodology in practice, e.g., when investigator(s) use(s) the available method-in-tools (i.e., descriptions, instructions, handbook, and tools) in a certain context of usage. Hence, these interdependent terms correspond to and converge with the purpose of accompanying the terms method-in-concept and method-in-action, which are mediated through using the available method-in-tool(s) (for further details, see [96]).

There is a lack of relevant literature on methods for evaluation method development within HCI, UX, and HRI, although the existence of these methods is found in large quantity. However, the general evaluation method development process presented by Blandford and Green [94] offers a tentative example of a kind of meta-method for evaluation method development within HCI that takes a human-centered approach. The motivations for using Blandford’s and Greene’s method development approach [94] for designing and developing ANEMONE are the following. Blandford and Greene are prominent scholars within the HCI community, and together, they have extensive experience and skills of developing several evaluation methods. They have been directly involved as creators in the development and testing of more than ten evaluation methods within the field of HCI throughout the years [76,94]. Their proposed method development approach is the outcome of these endeavors and their personal reflections upon that journey. We decided to apply their method development approach based on their experience and skills of developing and testing evaluation methods within HCI, the perceived soundness of their approach, and our prior usage of it in another context [55]. In addition, we wanted to be transparent to the readers regarding how we developed the ANEMONE approach, because many evaluation methods seem to have emerged in a void, ad hoc, or through a strawman approach.

Blandford’s and Green’s evaluation method development process contains five phases that could be viewed iteratively: (1) identification of an opportunity or need, (2) development of more detailed requirements, (3) matching opportunities, needs, and requirements, (4) development of the method, and finally (5) testing of the method [54]. However, they explain that a method project does not have to cover all five phases of the process. As inspiration for the work carried out in this paper, we have mainly focused on phases 1–4, and the fifth one, testing of the method in practice, is carried out in an additional upcoming paper. Hence, in many ways, method/methodology development can be seen as an iterative design activity and is viewed as such in this paper. Blandford’s and Green’s [94] evaluation method development process can be considered in the light of Lings’s and Lundell’s [96] terms, as a way of clarifying the different steps of method/methodology development. In the beginning, it is more focused on the method-in-concept (phases 1–4), which for obvious reasons includes method-in-tool (phases 2–4), and finally method-in-action (phase 5), but not as straightforward as described here, because of the iterative nature of design and development.

The first phase, *identification of an opportunity or need* focuses on the identified need and motivation portrayed in the introduction for such a methodological approach as ANEMONE, in order to systematically provide guidance on how to evaluate the mutual recognition of actions and intentions between humans and robots from a UX view. Thus, the aim and intended benefits of the methodological ANEMONE approach are clearly defined [94,95]. An additional, indirect and intended benefit is to highlight and combine frameworks and evaluation methods that have theoretical and methodological validity, reliability, and practical applicability to the field of HRI.

The second phase, *development of more detailed requirements*, focuses on when and how ANEMONE should be applicable and used in practice. Blandford and Green [94] present some criteria for human-centered evaluation methods, and we centered on the following ones [94,95]:

- Scope: The scope concerns the clarity towards the intended investigators in regard of what kind of aspects and issues the method focuses on or not. We argue that this is especially important for the end-users/investigators when choosing appropriate UX evaluation methods and subsequently reflecting on the pros and cons of the obtained results, when applying a particular method, in this case ANEMONE.
- Validity: The evaluation method should provide support to the end-users by correctly identifying potential issues while interacting with a robot. The method should maximize the identification of relevant usability and UX problems, and at the same time, it should minimize identification of trivial or non-relevant UX problems. As pointed out by Blandford and Greene [94], this aspect is particularly important for evaluation methods, and therefore, we pay additional attention to this criterion.
- Reliability: This concept denotes to what extent different users applying the same method will obtain similar results, which is of major importance for ANEMONE
- Insights derived: This criterion is aligned to what extent a method’s obtained results provide credible insights in order to improve the interaction and design of a robot. In other words, will the outcome of the evaluation method of ANEMONE support the future development of fluency in the human-robot interaction from a UX perspective?
- Feasibility of encapsulating a theory: This concept considers the extent of feasibility of the underlying theoretical framework for the method, as well as serving as a means for encapsulating a certain theory. In our work, ANEMONE relies on activity theory (AT), UX evaluation methodology, and insights derived from embodied cognitive science.

Continuing on the above discussion, Nilsson [97] highlights several criteria that one should reflect on generally when it comes to method assessment. Firstly, she mentions that a method could be more or less *structured*, by offering procedural knowledge about its usage to the intended end-users/investigators. She emphasizes that a method could be used as an aid to structure the procedure and to organize tasks, activities, and results. However, it should be pointed out that it is important to remember that a method is not a sort of simple recipe to obtain successful findings, and therefore, a method should be applied with good judgment [97]. Accordingly, a more formal and structured method can hinder new insights, ideas, and findings since the scope is more rigid and pre-defined. Secondly, the *effectiveness* of a method is dependent on its ability to achieve the intended and promised outcome, in terms of its purpose and goal, which is related to validity and scope. Thirdly, the *construct validity* refers to the acceptability of the underlying theoretical foundations of the method, which is related to the origin and purpose of the underlying theoretical framework of the method (i.e., similar to *feasibility of encapsulating a theory*). The AT framework has a strong impact on ANEMONE (see Section 3.1 and 4). Finally, the concept of *reliability* refers to the repeatability of the obtained results and findings when investigators use the method in practice, i.e., method-in-action [94,95,96]. In ANEMONE, we focus particularly on *structure* and *construct validity*. As starting points, the generic UX evaluation methodology (e.g., [61,63,64,65,71] was selected for *structure*, and AT (e.g., [81,82,83,84,85,86]) was selected for *construct validity*.

The motivation for an UX evaluation approach is the increasingly received attention of UX in HRI, recognized as vital for the proliferation of robots in society beyond the restricted zones for robots such as in an industrial setting [1,4,5,51]. Several issues that refer to the social and emotional aspects of the *interaction quality* between human(s) and robot(s) have earlier been focused on in the HRI literature, in which factors like engagement, perceived safety, intentions, acceptance, trust, cooperation, feelings, emotional response, likeability, and animacy have been studied [6,9,10,98]. We emphasize that when envisioning as well as embarking on such an approach, a lot of inspiration and lessons learnt could be obtained from the UX and HCI fields, which since the mid-1980s have performed systematic evaluation of various kinds of interactive technologies from human-centered and UX perspectives, even before robots entered the scene (e.g., [99,100,101,102]).

The motivation for using AT is based on the view that developing useful robot technologies should not focus on the technology *per se*, but address the need of understanding and knowledge of how potential users make sense of the robot technology in the particular context. AT fits hand in glove for that above addressed need, with its focus on understanding and describing human activity in context, i.e., stressing that the human mind can only be understood in the context of human interaction with the world, and this interaction, i.e., activity, is socially and culturally determined [81,82,83,84,85,86]. Bannon [77] was influenced by AT when he coined the phrase “from human factors to human actors”. It should be highlighted that AT has been acknowledged as one of the most valuable theoretical frameworks for HCI and tentatively for UX by many researchers, especially for its focus on agency by acting through technology and the social context of technology usage [76,81,82,83,84,85,86,88,103,104].

The third phase in Blandford’s and Green’s [94] process, *matching opportunities, needs and requirements*, considers the exploration stage, in which the following question could be asked; how should the identified need be addressed or are there any unidentified needs that could be fulfilled? In this phase, we performed research on existing methods and theory within the focal area of the identified motivation and needs with the aim to identify relevant existing and neighboring methods and models that could be suitable for application, modification, or inspiration as well as identifying theoretical frameworks that could be used as a lens in the development of a method [94].

In the work leading up to this publication, we realized that we had to narrow down the more generic UX evaluation approach combined with AT as the theoretical lens by paying more attention to actions and intention recognition *per se*. One tentative way forward in this step was to take a cognitive science-oriented approach, since some prior research on action recognition between human(s) and robot(s) has investigated and analyzed how social robots could be used as “interactive probes” to study and assess the specific sensory and motor mechanisms that are used in social human-human interaction (e.g., [17,18,19,20,24,26,28,30,31,32,33,34]). Related research has been carried out in order to investigate whether a similar ‘understanding’ could also happen beyond interacting humans to encompass interactions between human(s) and robot(s) [30,31,32,33,34]. In other words, it has been investigated whether and to what extent robots could be perceived as goal-oriented agents by humans. The obtained experimental findings reveal that there seem to be a comparable non-conscious processing of humans’ as well as robots’ actions, although more research on this topic is much needed. Some social robot researchers propose to use human’s anticipatory gaze behavior of robot action as a tool for evaluating the interaction quality between humans and robot(s), which is also a promising approach [31]. However, this more experimental cognitive science-oriented approach was not well-aligned with the overall purpose and scope of ANEMONE, and therefore, the intentional system theory by Dennett [58] provided a pragmatic way for HRI to deal with this matter. As a result, we took a closer look at what the HCI domain could offer. In HCI a wide range of cognitive theories and models have influenced and been applied in the field [76]. One of these is Norman’s [91,105] well-known seven stages of action model, which offers building blocks of human actions in relation to the world, useful for analyzing the interaction between humans and a digital artifact in general. Although HCI and HRI share some similarities, they also differ since HRI has certain characteristics which also influence the empirical study and evaluation of interactions between humans and robots [60,61,62,63,64,65,66,67].

The fourth phase, *development of the method*, is in itself an iterative and explorative process similar to the iterative processes taken in all kinds of design. Furthermore, Blandford and Green [94] point out that this phase does not provide much support in terms of structured processes, although they emphasize that drawing inspiration from existing evaluation methods is a good point of departure. The majority of this paper presents the outcome of phases 2–4 (see Section 3 and Section 4), which to some extent is related to the fifth and final phase, i.e., *testing of the method,* which is of fundamental importance for methodology development as well as a methodology’s usage in practice, which corresponds to the method-in-action term. Consequently, Blandford and Green [94] created the PRET A Rapporter framework, in which six aspects are proposed to consider when developing and testing an evaluation method [94,95], which is briefly summarized below:

1. Purpose: What are the goals and objectives of the evaluation? What questions do we want to ask and be answered?
2. Resources: What resources are available for the evaluation and what kind of constraints, e.g., time, knowledge, financial support, and skills, do we have to take into account?
3. Ethics: What ethical concerns do we need to raise and handle properly?
4. Relevant data collection techniques have to be identified and selected.
5. The collected data must be analyzed via proper analysis approaches and/or techniques that should be identified and selected.
6. Finally, the obtained findings should be properly reported.

Inspired by Blandford’s and Green’s above framework and their overall evaluation method development process [94], we have tried our best to consider and had the intention to address the issues and criteria raised above. We have identified the need for the ANEMONE method as well as formulated additional motivations for it. We have raised and considered ethical aspects when doing UX evaluation. We have also identified relevant data collection techniques as well as provide detailed guidance on how the collected data should be analyzed, in order to identify UX problems and then organize their severity and scope (see Section 4). The reporting of findings is aligned to the ANEMONE method as portrayed in this paper.

In this paper, the testing phase of the ANEMONE is divided into two sub-steps. The first sub-step consists of a validation of the underlying theoretical and methodological foundations of the UX evaluation and its relevance for the intended users of ANEMONE. The second sub-step refers to the testing of the evaluation method’s usage in practice (which refers to method-in-action). In this paper, we focus on the former, which refers to the concepts of method-in-concept and method in-tool. A series of workshops were arranged with the goal to discuss the need for this kind of evaluation methodology, its feasibility, validity, learnability, and underlying theoretical concepts. The method-in-concept and method in-tool of the ANEMONE methodology evolved between the workshops, because input from participants was considered in the subsequent versions of ANEMONE. The participants in the workshops ranged from cognitive scientists, cognitive psychologists, roboticists, human factors specialists of autonomous vehicles, engineers, auto safety researchers, computer scientists, UX designers, to human-robot interaction specialists. Each workshop lasted between one to three hours. A total of 5–15 participants were involved in each workshop. In addition, one human factors specialist in autonomous vehicles and one product development engineer provided detailed feedback based on their competence and the method’s applicability for the intended investigators and users of the ANEMONE approach. The workshops were performed in the context of two larger research projects that focused on various kinds of autonomous intelligent systems including social robots, autonomous vehicles, and human-robot collaboration. (For further discussion on the reflections upon using Blandford’s and Greene’s approach as such and for the insights provided for the design and development of the ANEMONE approach, see the Discussion, Section 5.2).

## 3. Theoretical Lens and Methodological Foundations

This chapter presents in more detail the basis of ANEMONE, having a foundation in AT, the seven stages of action model, and user experience evaluation methodology, which together are useful in motivating and framing the work presented in this paper.

### 3.1. Activity Theory—A Theoretical Lens

Activity theory (AT, or cultural-historical activity theory) offers a comprehensive conceptual framework that could be used for grasping and portraying the overall structure, development, and setting of *human activity* (which is a concept that we will characterize in more detail in Section 3.1.1). AT’s main focus is on the individual human being, the artifacts he/she uses, and other humans that participate in everyday activity, and how they are closely interrelated [80,81,82,83,84,85,86]. AT is based on thoughts and insights from a group of scholars, belonging to what nowadays is called the School of Russian socio-cultural psychology, in which Leontiev [82,106] and Vygotsky [107] are the two most prominent exponents. AT has been widely and successfully applied in human-technology/computer interaction research since the mid-1990s [76,80,81,82,83,84,85,86,103,104,108,109], and it has been stressed that AT is the most canonical theoretical foundation for HCI research [86]. As pointed out by Kaptelinin and Nardi [84], AT enables a relevant way of framing the human-technology interaction within a meaningful setting, i.e., the *context*, and provides several ways to gain a better grasp of the mutual ways in which technology affects—and conversely is affected by – the individual human being and groups of individuals. Furthermore, AT enables the possibility to clarify the underlying meanings of human’s usage of technology in general. It is argued that the application of AT for studying general human-technology interaction could be referred to as a symbolic shift in conducting research, from emphasizing the technological innovations to highlighting the various ways human users interact with technology as well as with each other, encapsulated within a theoretical framework that is governed by specific prerequisites and limitations [77,78,79,80,81].

In robotics, AT has been used to a limited extent as a general theoretical framework (but see [110,111]), although AT principles to various degrees are found in educational robotics (e.g., [112,113]), robot-assisted therapy (e.g., [114]), and developmental robotics (e.g., [25,115]. In this paper, AT is used as a basis for evaluating action and intention recognition in HRI from a UX perspective.

#### 3.1.1. The Concept of Activity

Central in AT is the notion of *activity*. What an activity is could be understood on an intuitive level, but in AT, “activity” is characterized as a purposeful, transformative, and developing interaction between actors (so-called “subjects”) and the world in the form of the actual context (so-called “object”) [84,85,86]. Hence, its focus is on understanding and describing human activity in context, i.e., stressing that a full understanding of human beings could only be carried out in the context of human interaction within the surrounding world and that this perspective on the human-world interaction, i.e., activity, is both socially and culturally regulated [104]. This socio-cultural dimension is stressed by the co-operative nature of human activity. As pointed out by Susi [116], to gain a deeper understanding of the individual human’s actions, these actions have to be viewed in the context of the overall collective human activity, because “[h]uman labor…is co-operative from the very beginning. We may well speak of the activity *of the individual*, but never of *individual activity*; only actions are individual” [117], cited in Susi [116] (p. 78). In other words, every activity consists of a set of intentionally performed goal-directed actions that could be described as an interaction between a subject (person) and an object (manufacturing site, public traffic, museum, airport) with the aim of transforming the object through the use of mediating artifacts like robots and additional tools like screws and hammers and traffic signs and so on. In so doing, individual humans have to be perceived of as active agents, who intentionally engage in artifact-mediated interaction processes [77,78].

#### 3.1.2. Basic Principles

AT is built upon five basic principles: hierarchical structure of activity, object-orientedness, tool mediation, internalization-externalization, and development. The first four are presented below as being the ones relevant for the purpose of the methodological approach to HRI evaluation presented in this paper.

##### Hierarchical Structure of Activity

**Figure 1:**
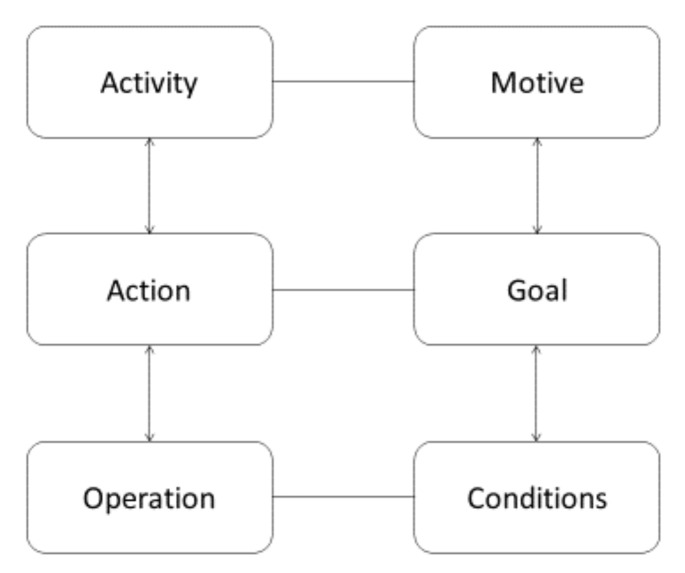
Activity levels (modified from [76,85]).

The first principle is the three-level *hierarchical structure of activity* that serves as the backbone of AT, framing consciousness at different levels. The levels are activity, action, and operation, which are related to motive, goal, and condition, respectively (see Figure 1) [76,85].

Briefly stated, the top layer is the activity itself, which is undertaken in order to fulfil some motive that corresponds to a certain need [76,83,85,116]. As phrased by Leontiev, behind a motive “there always stands a need or a desire, to which the activity always answers” [104] (p. 29). Actions (what must be done) are conscious processes subordinated to activities, and they are directed at specific conscious goals. These goals could be decomposed to sub-goals, sub-sub-goals and so on. Actions are similar to what usually is referred to as tasks within the HCI literature. Actions are implemented through lower-level units of actions, called operations (how it can be done), defined by the prevailing conditions or circumstances under which they are carried out. Operations are routine processes that do not have their own goals, instead they provide an adjustment of actions to the ongoing situations. Operations are oriented toward the conditions under which the subject is trying to reach a goal, and humans are commonly not fully aware of their operations. Operations could be the result of an automatization of a prior conscious action, which over time may be transformed into a routine operation that may not require conscious control. However, when operations fail, they are usually transformed into conscious actions again. Operations are often transformed from actions, although actions involve explicit goals that are conducted consciously initially. However, over time and pro-longed practice they become transformed into operations through the process of learning [76,80,81,82,83,84,85,86,116,117]. An example used by Leontiev [106] to explain the difference between *activities*, *actions*, and *operations* is how to master changing gears when learning to drive a car with a gear stick, portraying the dynamic nature between these concepts. The overall motive here is to obtain a driving license by learning to drive a car, where changing gear while driving the car is a fundamental activity in the beginning. The focus on hand-foot coordination when changing gears requires the full conscious attention of the learner initially, perhaps at a non-crowded parking lot. The new activity is then altered to drive the car safely in public traffic, and changing gears is now considered an *action* that is performed consciously because the driver is still an unexperienced driver, but rather soon, as an outcome of the practice of changing gears, the acts of moving the clutch and gear stick are altered into *operations* that now are performed rather effortlessly. In the long run, the skilled driver has acquired the skill of changing gears, and therefore, driving the car is no longer considered as an *activity*; it is considered merely as an *action*, being a part of another activity, e.g., getting to university or going to visit a friend’s house [82,106]. This means that the *action* of changing gears has altered to a routine *operation* that does not require any conscious effort for the driver in this moment. In other words, an *activity* has a dual character because it both mediates as well as is mediated by the physical and psychological tools that are used, which are situated within the particular social and cultural context of use of the activity. Changing the context to human-robot collaboration could be described as follows. An assembly worker in manufacturing has the motive to collaborate smoothly with a robot at the workplace (activity), and the needs to be fulfilled are to reduce the strain of the lower back; meanwhile, the collaboration with the robot should be perceived trustworthy and natural. The fundamental activity in the beginning is to perceive the robot’s actions and intentions during the task allocation between them when mounting a heavy part on an engine. The new form of collaboration requires the full control of the worker to interpret the movements of the robot as well as recognizing the intentions of the robot in the particular assembly task. After a while, the new activity of collaboration starts to run more fluently, and the worker can assembly rather smoothly with the robot in a rather slow pace. After prolonged practice, the task allocation in the assembly of the engine part with the robot becomes an *operation* that is performed effortlessly. There are complex relationships between motives and goals, which is the result of a complex social organization of human life. Viewing human activity as a three-layer system offers the possibility for doing a combined analysis of motivational, goal-directed, and operational aspects of human activity in the socio-cultural and material world, by interrelating the issues of “why”, “what”, and “how” within a coherent framework [76,80,81,82,83,84,85,86,116,117].

##### Object-Orientedness

The second principle, *object-orientedness*, is related to the hierarchical structure of activity. This principle is directly related to the very concept of activity as a subject-object relationship and, according to Kaptelinin [86], has similarities to the concept of intentionality. Generally speaking, the subject is the doer, the object is the thing being done with, and whom is the subject, and what is the object depends on the activity itself. Object-orientedness means that activities are always directed towards an object and each activity is distinguished from others according to the different objects [116]. Kaptelinin [86] points out the language problem when doing adequate translation of Leontiev’s notion of “object” from Russian to English. In Russia there are two words which have similar but separate meanings. The Russian notion refers to “predmet”, which implies that objects are objectively existing entities that usually have relevance to certain human purposes and interests. Roughly speaking, objects could be described as various settings in the world, e.g., a farm, a school, a factory, or public traffic. The connection to intentionality or aboutness is emphasized by Leontiev [82] who states: “[i]t is exactly the object of an activity that gives it a determined direction” and “the object of an activity is its true motive” [116] (p. 80). This means that behind the activity there is always some need to be met, and the motive may be material (human or a physical) or ideal (mental) objects.

Thus, the principle of object-orientedness declares that all human activities are directed towards different objects (e.g., public traffic, a manufacturing site, a nursing home for elderly, airport) and these objects motivate and direct activities. Activities are coordinated around objects, and thus, analysis of objects is necessary for understanding human activities, both at the individual and collective levels. In other words, the principle of object-orientedness stresses the actual setting and context of human interaction with the world, in which mediating artifacts and target tools are involved in activities [115]. It comprises the environment of the use of the target technology, e.g., the intended purposes and ways of using robots could only be understood within the current context of usage. In other words, object-orientedness refers to the *current context and setting of usage*, where the human (subject) interacts ‘indirectly’ with the context (object) through a mediating tool/artifact that could be a robot.

##### Tool Mediation

The third principle of AT emphasizes social factors, the focus on interaction between humans and their environment, and clarifies why the principle of *tool mediation* is the core of Russian cultural-historical psychology, having roots in Vygotsky’s seminal work [15,25,85,86,107,116]. The concept of mediation, i.e., the tools that mediate our actions, is strongly related to object-orientedness. The tool concept in AT is broad, and it embraces both material, physical tools (e.g., hammer, computer, phone, screws, ruler, calculator, camera, map) and psychological tools (e.g., signs, symbols, language, procedures, rules, methods), shaping the ways humans interact with the world.

The socio-cultural dimension of tool mediation is stressed by the fact that mediation enables various developed forms of acting in the world. The mediating tools we use, modify, and develop incorporate the accumulation and transmission of social and cultural knowledge in a society. This means that the tools we use in our everyday activities become transformed, and the historical development of an artifact (e.g., computer, robot, traffic rules) is crystallized into the artifact/tool itself [116]. However, it should be pointed out that the use of tools not only transforms the objects themselves, rather it is a mutual ‘two-way process’, where tools reflect previous experiences of using the tool as well as how to design the tools, i.e., tools embody a set of social practices and their current design reveals a history of particular usage (e.g., the design and development process of steering wheels and blinkers from early cars to today’s self-driving cars). In other words, a robot can be considered as a mediating artifact, and the ways we interact with a social robot through gestures, gaze-following, or verbal utterances are also considered tools. The way that the mediating artifact responds to us also influences our perceptions of it in a dual process.

It should be stressed that a robot is typically not an object of activity but rather the main mediating artifact/tool [86]. Instead, the object of activity is the actual setting and context in which the robot is used (manufacturing site, nursing home, classroom, public transport, or airport). This means that humans *per se* are not interacting with robots, but interact with the world through them. This means that we should not be conducting research on human-robot interaction, but rather studying robot-*mediated* interaction. Bødker [118] stressed the issue in the early 1990s, and this perspective on interactive technologies situates computers as well as robot usage, although not explicitly addressed by Bødker, into AT’s hierarchical structure of human activity, aligning the operational aspects of the interaction with interactive technology like robots to meaningful goals and, in the long run, the needs and motives of the users of the technology [86]. The robot as a mediating artifact can be exemplified at an assembly station where it should support the human operator in avoiding heavy lifts so he/she feels comfortable at work. A social robot at the airport (context/setting) should help us find the shortest way to the gate so we do not miss the connecting flight. The socio-cultural dimension of tool mediation offers possibilities to analyze the context and practice of technology usage, and AT provides several concepts and principles for considering the design, development, evaluation, and deployment of interactive technologies (e.g., [76,108,119]). In other words, *tool mediation* emphasizes that a tool like a robot comes fully into being when it is used in a particular context and that knowing how to use it is a vital function of the robot.

##### Internalization-Externalization

The fourth principle of AT is *internalization-externalization*, which stresses that human activities are dynamically distributed between the external and internal side [15,25,83,86,104,116]. The traditional notion of mental processes corresponds to internal processes only, but AT does not accept a dualistic conception of an inner isolated mind and an outside world [83]. AT emphasizes that the internal side of an activity cannot exist or be analyzed in isolation, without the external side. Hence, an activity has a dual nature, because every activity has both an external and internal side. Internal human activities integrate the experience of human beings in the ways they are manifested in the corresponding concrete and practical (external) activity [83]. It should be acknowledged that the internal and external sides of activity are being gradually more intertwined in human work and life [15,25,83,86,104,116,120]. Roughly speaking, the *internalization-externalization principle* is the ongoing shifting back and forth between what happens internally “in the head” and what happens practically and externally “in the open” in human activity, being two sides of the same activity coin. In other words, the internalization-externalization principle could be described as a cycle of interactions being both physical ones, like performing a greeting action with your hand (external) towards the social robot with the intention to gain its attention. Then, your own (internal) interpretation whether the robot ‘understands’ the motive of the greeting gesture, which could be that the robot pays its attention to you by turning towards you (external), which you may interpret (internal) as yes, it did.

In sum, the above presented basic principles of AT consist of an intertwined system forming a whole that represents several aspects of human activity, resulting in the activity being the minimal meaningful unit to study. This addresses the need to systematically apply these principles from a holistic perspective, because of their interrelatedness, which unfolds over time, i.e., the *developmental principle* that has not been explicitly presented here. The developmental principle stresses, among other things, how tools and the usage of tools unfolds over time in their context of use, instead of only testing them once in contrived settings.

#### 3.1.3. The Activity System Model

**Figure 2:**
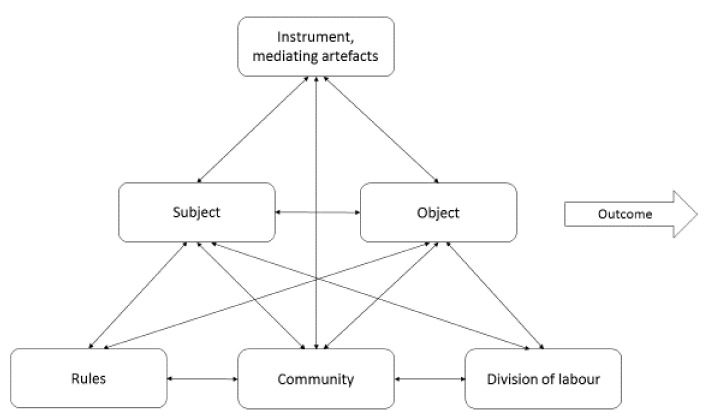
The activity system model (ASM)—Engeström’s triangle (modified from [117] (p. 63)).

Engeström’s activity system model (ASM) [117] is an elaboration of AT to further encompass the community level (see Figure 2). The interactions between subject, object, and community are mediated by specific kinds of mediational means, including *mediating artifact* and *tools/instruments* for the subject-object interaction (from Leontiev’s [106] original work). Beyond these above interactions there are *rules* that regulate the subject-community interaction as well as the *division of labor* that regulates the community-object interaction. The ASM also includes the *outcome* of the whole activity system, which is the obtained result from the transformation of the object (world) that is being altered by the core activity. The outcome of the ASM could then be served into another ASM [86,117]. It should be pointed out that Engeström’s [117] view is based on a holistic systems approach to conceptualize humans’ intentional activities, rather than considering humans as passive factors lacking any internal properties or motives (see the principle of externalization/internalization). This way of thinking highlights the continuous process of transformation and development over a time horizon.

Kaptelinin [86] points out that investigating and analyzing complex real-life phenomena via the activity system model, often requires networks of activity systems models, because analyzing one activity system model is not sufficient. A central part when analyzing an activity system is the search for so-called *contradictions* within the system, i.e., any misfit within an element in the system, between elements in the system, or between the current activity system in relation to other activity systems to which it is linked [117]. These contradictions are commonly manifested as problems, interruptions, workarounds, or breakdowns. When developing a collaborative robot, the outcome of the interface designers’ activity system should be integrated with the outcome of the robot developers’ activity system. Several trade-offs have to be made between these activity systems when evaluating a new gaze-following mechanism to be used for action and intention recognition in the task-allocation between the human and the robot, in order to develop an intuitive user interface so operators could interact smoothly with the robot, to fulfill their needs and motives. In AT, these contradictions are usually considered as sources of development, because human activities are often work in progress to handle these contradictions [83,86,117]. Therefore, it is of major importance to study contradictions from several perspectives, shifting focus from the actions of the individual to zooming out to the broader activity context and then zooming in again. When studying self-driving cars, the researchers can shift the focus from the individual driver/passenger to the stream of cars crossing an intersection. The purpose is to gain a better understanding of the nature of the contradictions. Engeström [117] denotes these developmental cycles, in which contradictions are the driving force, as expansive cycles. Engeström’s [117] focus on development in his ASM emphasizes how work practice and other actions are constantly evolving, they never stand still, and a change of tools/instruments changes work practice, and the changes in work practice reshape the tools/instruments that are used [79,80,81].

#### 3.1.4. AT as an Analytical Lens

Engeström’s [117] activity system model has highly influenced the fields of HCI, computer-supported cooperative work (CSCW), education and design and systems development [76,84,85,86,103,104,119,120]. The activity system model (ASM) has been used to analyze varying kinds of work settings, particularly when there are problems with current or newly implemented technology and where the ASM enables both micro and macro level issues to be identified [119]. A common approach to frame the analysis of the activity system model is to use the approach developed by Mwanza and Engeström [121], which commonly is known as the eight-step model. Their eight-step model provides a structured way to characterize the activity and sub-activity triangles in the activity system model. The first step is to describe the activity under investigation. The second step is the asking of the “why” motive behind the activity. The third step is to identify the actors (subjects) who perform the activity in the first step. The fourth step is to identify the mediating artifact and the tools that mediate this activity. The fifth step determines the rules that constrain and regulate the activity (it should be pointed out that Engeström used the concepts “mediating artifacts”, “instruments”, and “tools” rather interchangeably in his writings). The sixth step tries to understand and describe how labor is divided and distributed among the actors (subjects) who participate in the activity system. The seventh step is to explain the community of actors involved in the activity. The eight step is to identify the expected outcome of the activity. As pointed out by Mwanza [122], defining and characterizing the elements of the ASM provide some support for the analysts when answering the eight questions, which focus on answering several issues about the interrelations between the components within the ASM.

Halverson [119], among others, suggests that theories like AT could be viewed as conceptual tools for making sense of a domain. AT provides help in asking the right questions rather than providing ready-made answers [76,86,104,119]. Furthermore, Rogers [76] points out that although AT has rhetorical power, its success in usage still relies basically on the analysts’ own skills in interpreting and orienting themselves in the course of analyzing the collected data and to properly relate the data to the concepts of AT. There is limited guidance on how to do this important work in AT, especially in identifying and determining the different levels of activity.

One of the largest problems addressed in applying AT is to identify the proper level of activity [76], because it is not easy to reveal and explicitly articulate the human’s motives. A tentative way forward to overcome this issue is to start focusing on the action level, but this is more easily said than done. To reduce this problem, we suggest that Norman’s [91,105] seven stages of action model provides a tentative way to find the appropriate level of activity for ANEMONE. Thus, our suggestion is to use Norman’s model in order to identify the proper level of activity in relation to the three-level hierarchical structure of activity, i.e., linking it to the action level (see Figure 1).

### 3.2. The Seven Stages of Action Model

The seven stages of action, as called by its creator Donald Norman [91,105], is an approximate model and not a complete theory of action. It provides a structure of fundamental building blocks of actions that is useful for analysis of the interaction between humans and the world (object-orientedness), specifically human-digital artifact interaction, with the human at the center of attention. It should be emphasized that Norman [123,124,125] is strongly influenced by AT’s hierarchical structure of activity [123] and the dual aspect of tool use [123,124,125,126], although this is not explicitly stated in the original seven stages of action model [91,105]. Norman addresses that human-centered design needs to be expanded into activity-centered design, arguing that “successful devices are those that fit gracefully into the requirements of the underlying activity, supporting them in a manner understandable by people. Understand the activity, and the device is understandable” [123] (p. 15).

There are also several similarities in Norman’s [124] view on artifacts as mediators between humans and the world (object) via feedback flows in the continuous action cycles that constitutes the nature of the interaction with Engeström’s [117] expansive cycles, focusing on going from the more abstract to the more concrete, in specific epistemic actions. Taken together, this exemplifies dialectical-theoretical thinking, capturing the smallest unit of analysis, which together forms a cycle or spiral of epistemic actions in an expansive cycle of a functionally interconnected system [117].

The seven stages of action model has been proposed for the HRI field by others, e.g., Scholtz [127] and Taha et al. [128], although not as basis for UX evaluation of action and intention recognition. Based on AT’s influence on Norman’s work [123,124,125], we interpret the seven stages of action model as being more aligned with an AT approach than a classical information-processing approach of human-computer interaction [76,77]. Arguments for our AT inspired view of Norman’s model is the focus of *actions*, being aligned to the basic principles of hierarchical structuring of activity, tool mediation, object-orientedness, and externalization/internalization (see Section 3.1.1 and Section 3.1.2).

**Figure 3:**
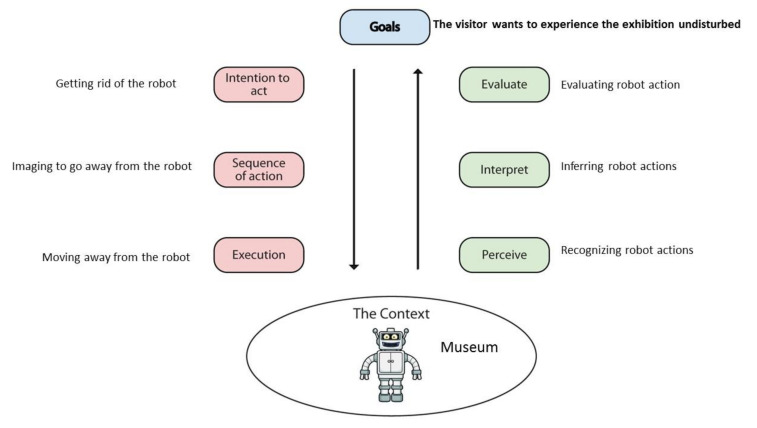
The modified seven stages of action model including a robot (modified from [91,105,124]).

According to Norman [123,124,125], the action cycle should be considered in its *context of use*, and therefore, we have changed the original concept of the word to the concept of context in our modified version, to be more aligned with the AT. The initiation of action can either begin in the mind of a person or as a reaction to a state of something in the surrounding context (object), i.e., an action can be either goal-driven or event-driven (also called data-driven), and originates from the overall motive (activity) of the user [91,105,123,124]. As emphasized by Norman [105,123], actions are in fact much more complex than the simplified model implies. Thus, an activity can consist of many nested loops of actions, stages can be skipped or repeated, and motives and goals can change. Prolonged practice may result in the specified sequence of actions being transformed to automatized operations. The stages of the Norman model are described below and depicted in Figure 3, in which we have modified the original model as well as included a robot (mediating artifact):

1. *Forming the goal*: there is a gap between the current and the desired state that the person wants to overcome.
2. *Forming the intention to act*: a plan to do something to overcome the gap is shaped and a direction for how to achieve it is outlined,
3. *Specifying the sequence of action*: the details on how to carry out the chosen “strategy” is formulated.
4. *Executing the action*: physically executing the specified sequence of action.
5. *Perceiving the state of the context (object)*: noticing the things that can be sensed.
6. *Interpreting the state of the context (object)*: conceiving what the sensed things mean.
7. *Evaluating the outcome in relation to the goals*: comparing the new state with the desired one and assessing if the goal is fulfilled to enough extent.

The path going from the current situation in the context to the goal stage is termed *gulf of evaluation*, representing the comprehension gap between the actual state of the robot in its context and the goals and overall motive of a person. This gulf is handled by the human by making sense of what can be obtained from the robot in relation to the desired state/situation in the context [91]. This means that the gulf of evaluation refers to the difficulty of assessing the current state/situation in the context and to what extent the robot supports the detection and interpretation of that state in the particular context [124]. Imagine a humanoid robot at a museum (context) with the purpose of guiding visitors through an exhibition. A visitor enters the room where the robot is located. The robot turns its face and body towards the visitor, which is observed by the human (perceive—recognize action). The visitor believes that this action means that the robot’s intention is to approach him/her expecting to communicate in some way (interpret—infer intention). The visitor is not in the mood for interacting with the robot, instead he/she wants to stroll around in the exhibition without being disturbed (evaluate).

The other path from the goal stage to the situation in the context is called *gulf of execution*, in the sense that there is a gap between the desired and the actual state of the robot (mediating artifact) in its context (object). Humans manage this gulf by doing something to the robot or taking other measures in relation to it [91]. This means that the gulf of execution refers to the difficulty of acting upon the current situation in the context and to what extent the robot supports those actions [124]. To continue the example with the robot at the museum; the visitor wants to experience the exhibition undisturbed (goal) and would like to get rid of the robot (intention to act). The visitor imagines that moving away from the robot (sequence of action) is a proper way to achieve this goal and accordingly does so (execution). The visitor glances furtively over the shoulder at the robot, then, noticing a change in direction of the robot’s head and body (perceive), infers the robot’s action as it no longer intends to interact with them (interpret). The visitor is relieved thinking that now he/she can enjoy the exhibition on his/her own as originally intended (evaluate). Norman [91,124] points out that there exist two ways of bridging the above gulfs: either by suitable design of the artifact or via cognitive effort and practice. Through prolonged practice, the person develops skills to handle the artifact, and subsequently, the person cognitively bridges the gulfs so that the actions upon handling the artifact are performed subconsciously, being transformed to operations. This may result in the situation where the person considers himself/herself to act upon the world (object) directly, because the artifact ceases to ‘exist’ anymore (see Section 3.1.2)

By addressing each stage in the action model from a human perspective in terms of interacting with the mediating artifact in the form of a robot, it is possible to analyze whether the robot provides proper cues and information for the human to act on. For instance: is it possible for a person to specify a suitable sequence of action based on the interface of the robot? Is it easy to physically carry out the needed actions? Is it possible to notice the state and/or mode of the robot? Can the user draw correct conclusions between the interpreted state of the robot in its context in relation to his or her own goals and motives? A robot that functions properly in the mediation between the human and the surrounding context results in what Bødker [118] denotes activity flow. The concept of activity flow is described as a smooth and somewhat ‘automatic’ activity cycle in accomplishing the goal. The resulting positive experience of smooth, fluent, or natural engagement appears when a cohesive activity flow is supported by the task, the artifact, and the surrounding context, without any unexpected outcomes or interruptions that hinder or break down the activity flow [118,125]. Thus, activity flow has a positive impact on user experience of interacting with the robot.

However, the seven stages of action model is too limited to provide the foundation for a proper evaluation methodology of ANEMONE, and therefore, the next section describes a brief insight into UX evaluation methodology.

### 3.3. UX Evaluation Methodology

The UX concept can be experienced and interpreted rather vaguely, but the ISO’s specification on ergonomics of human-system interaction [70] (clause 2:15) provides some clarification, and defines UX as “a person’s perceptions and responses that result from the use or anticipated use of a product, system or service. Note 1 to entry: User experience includes all the users’ emotions, beliefs, preferences, perceptions, physical and psychological responses, behaviours and accomplishments that occur before, during and after use. Note 2 to entry: User experience is a consequence of brand image, presentation, functionality, system performance, interactive behaviour and assistive capabilities of the interactive system, the user’s internal and physical state resulting from prior experiences, attitudes, skills and personality, and the context of use. Note 3 to entry: Usability, when interpreted from the perspective of the users’ personal goals, can include the kind of perceptual and emotional aspects typically associated with user experience. Usability criteria can be used to assess aspects of user experience.” This means that, according to the above definition and notes, it is not possible to guarantee a certain UX, since it is the outcome of the subjective inner state of the user. Indeed, by carefully and systematically designing and evaluating the artifact for a high quality interaction with the intended users and the usage context in mind, it is possible to have a positive impact on the user’s experience [60,67,69,70,71,72,79,81]. As pointed out in the Introduction, there is still an existing lack of theoretical frameworks in understanding user experience [88,89,90], and of which AT has been viewed as a tentative and promising candidate [79,80,81,83,88]. The concept of UX embraces pragmatic quality as well as hedonic quality [71,81,129]. On the one hand, pragmatic quality is concerned with the usability and usefulness of the product/artifact/technology, i.e., the interactive product should enable the human user to accomplish the task-related goals in an effective, efficient, and secure way of working. On the other hand, hedonic quality is concerned with the psychological and emotional needs of human users that should be addressed by the interactive product. The human user could, e.g., perceive the social robot as being cool, amazing, splendid, human-like, trustworthy, satisfying, or enjoyable. The social robot might induce feelings of autonomy, competency, empathy, and kinship [61,64,71,81,129,130]. Like all other interactive products/artifacts for human use, users’ interactions with and perceptions of social robots evoke feelings of a different nature and intensity [60,61,62,63,64,65,66,67]. We agree with Savioja et al. [79] and Grundgeiger et al. [81] that UX to a large extent is a subjective phenomenon, which is deeply situated and embedded in the activity concept as characterized in AT. Therefore, we emphasize that AT is a very promising approach to illustrate the understanding of a user’s experience of grasping the actions and intentions of a robot while interacting with it, which is fundamentally a cognitive ability derived from the users’ needs and motives, which are in turn consecutively enacted by the social-cultural context in which the user is situated within the world.

As described by Hartson and Pyla [71], among others, the foundation of the UX design lifecycle process entails four central interactive phases that are called *investigate/analyze*, *design*, *implement*, and *evaluate* [64,65,71]. The UX design lifecycle process starts with the investigation and analysis phase, which aims to bring an understanding of the needs, goals, meanings, and emotions associated with the user’s work or other kinds of activities as well as the business domain of the interactive system/product/service. During the investigation and analysis phase, it is also important to identify and describe the technical and infrastructural constraints as well as the business’ and users’ needs [71,72]. Already in this initial phase, it is necessary to consider the user goals, which have to be linked to the business goals, and the business goals should be related to user behaviors [131]. The intended users groups have to be identified and described early on, and they should be focused upon during the whole UX design lifecycle process. It is also essential to center on the product mission throughout the whole process [131]. The next phase in the UX design lifecycle process is the design phase, which involves the formation of the key concept of the interactive system/product/service, its interaction behavior, and the ‘look and feel’ of the envisioned system/product/service. In the following implementation phase, the main focus is on prototyping, which ranges from low-fidelity to high-fidelity prototypes of the envisioned interactive system/product/service. In so doing, it enables the realization of the different design alternatives that are created. In this paper, the main focus is on the evaluation phase, in which the refinement and verification of the interactive system/product/service often happens. It should also be pointed out that a central principle of the UX design lifecycle process is the iterative and incremental nature of it, which means that performing UX evaluation is a central and imperative part of it [71,72,131].

The concept of UX evaluation entails a wide set of methods, techniques, and skills that are applied and often combined in order to systematically identify how users perceive and experience an interactive system/product/service before, during, and after interacting with it. It should be pointed out, however, that several misleading terms are commonly used in the HRI literature (e.g., [64,65,75,132,133]), where the term ”user evaluation” is commonly used, rather than the more appropriate terms “usability evaluation” [93] or “UX evaluation” [60,61,62,63,64,65,66,67,71]. At first glance, the difference between the terms seems negligible. However, it should be highlighted that it is the users’ experience of interacting with the robot/system/product/service that is the main focus in the UX evaluation, which means that it is not the users themselves that are evaluated, but aspects of the *interaction quality*, which is a significant difference. Usability evaluation mainly focuses on pragmatic qualities, being the forerunner of UX evaluation, which typically covers both pragmatic and particularly hedonic qualities [71,134]. The application of UX evaluation methods has been revealed to be positive in the HRI field, although limited work has been conducted so far [62,63,64,65,66,67,134,135,136]. It should be emphasized that it is not a simple task to measure, assess, and evaluate the users’ perceived UX while interacting with a robot, because it is rather a subjective phenomenon, which is dependent on prior experiences, preconceptions, attitudes, skills, and competence. In addition, the users’ perceived UX is dependent on the context of use, and to complicate the issue further, it dynamically changes over time [64,69,71,72,129]. Therefore, to ensure that a UX evaluation is successfully accomplished, the investigator has to raise some general questions early on in the evaluation process. The investigator could be asking, e.g., what UX dimensions, UX factors, and UX evaluation methods should be used, and should target the UX evaluation for the specific area of interest [65,66,67,69,71,72,93], e.g., certain aspects of HRI such as if it is possible for human users to correctly and with experienced certainty recognize the actions and intentions of a robot. There exists a number of UX evaluation methods and techniques for collecting quantitative as well as qualitative data [64,65,66,67,71]. As Law and Sun [88] point out, there is a lack of viable analytic frameworks that provide a deeper understanding of the dynamics of the factors influencing UX. We emphasize that this in particular also applies to the HRI context, and therefore, we consider AT as a promising and tentative candidate for providing such an analytic lens.

### 3.4. Summary of AT as a Theoretical Lens, the Seven Stages of Action Model, and UX Evaluation Methodology

To summarize, there is a lack of viable analytic frameworks that provide a deeper understanding of the dynamics of the factors influencing UX [88]. We consider AT as a promising and tentative candidate for providing such an analytic lens in HRI. AT provides a conceptual framework for understanding and describing human-robot interaction within a meaningful and social context, providing opportunities to better grasp the mutual ways robots affect—and are affected by—individuals and groups, as well as elucidating the underlying meaning of robot usage for humans. The notion of activity is the minimal meaningful unit of study, and every activity consists of a set of intentionally performed goal-directed actions with the aim to transform the context through the use of mediating artifacts [82,83,84,85,86]. AT comprises five basic principles: hierarchical structure of activity, object-orientedness, tool mediation, internalization-externalization, and development. The hierarchical structure of activity is activity, action, and operation, which are related to motive, goal, and condition, respectively. Viewing human activity as a three-layer systems offers the possibility of conducting a combined analysis of motivational, goal-directed, and operational aspects of human activity in its context, by interrelating the issues of “why”, “what”, and “how” it is performed [76,82,83,84,85,86,106,118]. Object-orientedness stresses that the intended purposes and ways of using robots could only be understood within the current context of usage. This means that behind the activity there is always some need to be met, and the motive may be to fulfil physical or psychological needs. An activity both mediates, and is mediated by, the physical and psychological tools used. Robots are here viewed as mediating artifacts/tools in the context where they are used, i.e., manufacturing site, nursing home, classroom, public transport, or airport. This means that humans *per se* are not interacting with robots, but interact with the context through them. The internalization-externalization principle is the ongoing shifting back and forth between what happens internally “in the head” and what happens practically and externally “in the open”, being two sides of the same coin. The developmental principle stresses how robots and the usage of robots unfolds over time in their context of use. The activity system elaborates AT to the community level [82,83,84,85,86,116,117]. The search for contradictions is a central part in the analysis of an activity system, which can display any misfit within an element in the system, between elements in the system, or between the current activity system in relation to other linked activity systems [79,80,81,117]. These contradictions are manifested as problems, interruptions, workarounds, or breakdowns in HRI. These contradictions are viewed as sources of development, because humans often try to handle them. It is beneficial to study contradictions from several perspectives, shifting focus from the actions of the individual to zooming out to the broader activity context and then zooming in again. The developmental perspective is always present, due to the fact that the change of robots influences work practice and private lives, and the changes in work practice and private lives reshape the robots and other tools that are used in that context.

One problem to be addressed in applying AT is to identify the proper level of activity, because it is not easy to reveal and explicitly articulate humans’ motives [76]. Norman’s seven stages of action model [91,105] provides a tentative way to find the appropriate level of activity in relation to the three-level hierarchical structure of activity, i.e., linking it initially to the action level. Norman’s model provides a simplified structure for an action cycle of seven steps, which is useful for analysis of HRI, although not initially developed for that purpose. By addressing each stage in the seven stages of action model from a human perspective in terms of interacting with a robot, it is possible to analyze whether the robot provides proper cues and information for the human to act on. The path going from the current situation in the world to the intended goal stage is termed gulf of evaluation and refers to the difficulty of assessing the current state in context [91,105,124] and to what extent the robot supports the detection and interpretation of that state in the context. The path from the goal stage to the situation in the context is called gulf of execution and refers to the difficulty of acting upon the current situation in the context [91,105,124] and to what extent the robot supports those actions. A robot that functions properly results in an activity flow [118,124], which is a smooth and somewhat ‘automatic’ activity cycle to accomplish the goal. Thus, activity flow has a positive impact on user experience of interacting with the robot.

However, the seven stages of action model is, according to our interpretation, too limited to provide the foundation for a proper UX evaluation methodology in HRI. It is not possible to guarantee a certain UX, since it is the outcome of the subjective inner state of the user [60,67,69,70,71,72,79,81]. A positive impact on the user’s experience is made possible by carefully and systematically designing and evaluating the artifact for a high quality interaction with the intended users and the usage context in mind [71]. Accordingly, UX is to a large extent a subjective phenomenon [65,66,67,69,71,72,93], and therefore, we emphasize that AT is a very promising approach to illustrate the understanding of a user’s experience of grasping the actions and intentions of a robot while interacting with it, which is fundamentally a cognitive ability derived from the users’ needs and motives. UX evaluation represents a number of methods, techniques, and skills that are used to identify how users perceive a robot, before, during, and after interacting with it [64,65,66,67,71].

Next, we present the outcome of the underlying theoretical and methodological foundation for the methodological approach as presented in Section 3, with a description of ANEMONE and its procedure.

## 4. Description of ANEMONE and its Procedure

In this section, we present and describe the approach of evaluating humans’ possibilities to correctly and with certainty recognize the actions and intentions of a robot. Its central components are based on generic UX evaluation methodology (see Section 3.3), in which elements from AT (see Section 3.1) and the seven stages of action model (see Section 3.2) are included. The focus of the ANEMONE evaluation methodology approach is to determine whether and to what extent humans can perceive, understand, and predict the intentions and actions of a robot and provide relevant comprehension of why something works or not in a particular usage situation and context.

Although ANEMONE consists of a phase-by-phase procedure, it should not be viewed as sequential; instead, it is an iterative process in which it can be necessary and advantageous to revisit phases and sub-phases, e.g., the goal setting stage where it may be needed to change initial high-level goals into more specific and focused ones after a first exploratory evaluation cycle. Using the methodology on a particular case requires preparation, selection of evaluation type, planning and conducting the evaluation procedure, analysis, and suggested changes. These phases are further detailed in Table 1.

**Figure 4:**
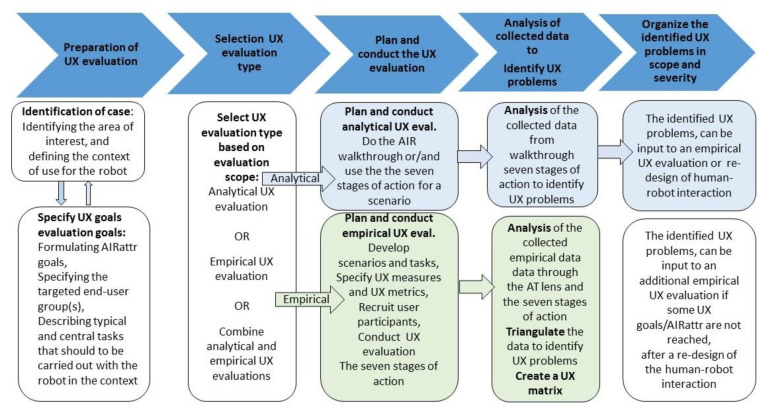
The overall description of the phases in the ANEMONE approach.

In practice, ANEMONE is used to sort out the overall procedure and selections made along the path from identification of the case by identifying the area of interest and defining the context to specifying UX goals, selecting the evaluation type, and planning, as well as conducting the selected evaluation and analyzing the collected data for the validation of findings, which results in suggested changes (see the methodology illustrated in Figure 4).

### 4.1. Phase 1: Preparation

A proper evaluation needs thorough preparation, which here means that the area of interest is identified, the context needs to be defined, and that the goals of the evaluation should be specified. This helps the investigator to focus on what is important, subsequently plan the evaluation procedure, and then analyze the interaction via the collected data.

#### 4.1.1. Identifying of the Case: Identifying Area of Interest and Defining the Context

Initially, it is necessary to identify the area of interest, i.e., identification of a case. The case could be using a service robot in a shopping mall or introducing a collaborative robot in assembly in manufacturing. These example cases of human-robot interaction are situated in a particular context, so there is a need to define the context in which the robot should be or is used. Human activity takes place in a social and cultural context, as strongly emphasized in AT (see Section 3.1), and so does the interaction between humans and robots. By carefully identifying the case and defining the context for the evaluation, it is easier to then investigate and analyze the main mediating artifact, which is the robot, identify the relevant instruments/tools involved in the target activities, and to constitute the environment in which the robot will be used to fulfill the human motives.

As pointed out by Bødker [137], design of technology must be based on applications in use within preferably empirical settings [79]. Accordingly, it is of major importance to situate the robot in a context where the robot will be used, preventing the investigator from considering the robot-mediated interaction in isolation.

#### 4.1.2. Specify UX and Evaluation Goals

For humans to achieve their motives and goals, they actively transform objects in their environment, to meet their psychological as well as emotional needs under current circumstances [1,34,35,36,37,38,40,41,42]. Studying the usage of any robot technology should benefit a deepened understanding and knowledge of how users make sense of technology. To better gain such an understanding, it is necessary to set several goals, against which the users’ experience of the robot usage could be evaluated.

The first issue that needs to be decided is what overall goals should be addressed in the evaluation [52,53]. When developing robots for real-world use in a particular setting, there probably are explicit or implicit goals for the robot as a product, for instance, regarding the market (e.g., elderly care sector) and general purpose (e.g., social companion) that create the frame within which the evaluation should be conducted. To define proper UX goals, it is also necessary to have a clear conception of the characteristics of the targeted end-user group(s), typical and central tasks that are to be carried out, and significant elements of the context [71,102,137,138]. The reason is that actual UX of an interaction is not absolute; it is always in relation to the user, task, and context. For instance, a certain alarm signal can be easy to perceive for a person with full hearing capacity, but impossible for a hearing-impaired person to notice. A robot can be an efficient navigation aid in a building, but be useless at identifying if a missing child is situated near it. In a neat space occupied by only a few people at a time, a moving robot can be experienced as safe enough, but in a crowded and messy space it can be viewed as a safety risk.

In an UX evaluation of action and intention recognition, the goals have to embrace UX-centered action and intention recognition (hereafter denoted AIRattr) specific attributes. On a generic level, the following high-level requirements need to be fulfilled in order to attain a foundation for the human-robot interaction to be experienced by humans as well-suited to its purpose, being smooth and satisfying to interact with.

- The robot needs to generate signifiers that humans can perceive.
- The meaning of the signifiers should be correctly understood by humans, in terms of predicting that the robot will do something and preferably also what it will do.
- The signifiers should be so obvious that the humans are certain of their interpretation of them.
- The meaning of the actions of the robot should be correctly understood by the humans.
- The actions should be so obvious that the humans are certain of their interpretation of them.

By rephrasing the seven stages of action (see Section 3.2), in this case initiated as a reaction to a state of the robot (in the form of the mediating artifact) or something else in the context (other tools/instruments including peers), this means that humans should, via ongoing externalization/internalization and focusing on the mediating artifact, correctly be able to:

1. **Perceive** the intention of the robot to perform an action, i.e., perceive the robot’s status in its context.
2. Predict what sequence of actions will take place, i.e., **interpret** the robot’s status in its context.
3. Assess the robot’s actions in relation their goals, i.e., **evaluate** the interpretation in relation the goals.
4. Determine if any measures need to be taken in relation to the robot in order to reach the goals, i.e., forming **intention to act**.
5. Specify a proper **sequence of actions** in relation the robot, i.e., understand and determine what it is possible to do in order to reach the goal.
6. Perform the chosen sequence of actions in relation to the robot, i.e., **execute** the sequence of actions.
7. Perceive the effects of the actions in relation to the robot’s status in its context, and interpret the effect, in order to **evaluate** the interpretation in relation the goals.

Together, the product goals, the information regarding the end-users, the task and the context characteristics, and the AIRattr requirements guide the formulation of high-level UX goals that direct the efforts and boil down to lower-level specified goals, which directs the design of the evaluation as a whole and in detail.

**Example**

- *Product goal*: A social robot at a shopping mall with the higher-level aim to improve the number of visitors at the mall and increase the amount of money spent in the shops.
- *End-user group*: Busy adults wanting to quickly and comfortably run their shopping errands.
- *Tasks*: Helping visitors to find what they are looking for and give relevant special offers.
- *Context*: Robot is located in the main entrance of the building, which is often crowded and noisy.
- *UX goal*: The robot should be experienced as polite and non-intrusive in the sense that a visitor clearly should be able to see that the robot is available to provide service and that he/she should feel that he/she is in charge of the situation.
- *AIRattr evaluation questions*: Is it possible for the visitor to perceive that the robot turns its face and body towards him/her? Is it possible for the visitor to correctly interpret what the turning of the robot means? Is it possible for the visitor to assess if the robot’s actions may be helpful in relation to their goal?

### 4.2. Phase 2: Selection of UX Evaluation Type

The next phase is to decide what type of UX evaluation is to be conducted. The decision is dependent on the defined evaluation’s goals, the robot’s developmental stage, i.e., from a conceptual idea or prototype to a completely functioning robot, and available resources for doing a UX evaluation (e.g., time, money, competence, equipment, usage context, and access to robot). If the resources are scarce and limited it is better to do some kind of minor analytical or empirical UX evaluation, although not as rigorously as a full-scaled empirical one, than none. It is also often better to carry out the UX evaluation, either analytical or empirical, as early as possible in the development lifecycle of the robot, because it is easier and cheaper to make changes to the robot than later on in the development cycle [63,64,71,138].

#### 4.2.1. UX Evaluation Scope

There are three interrelated layers of user needs involved in UX evaluation: the ecological layer, the interaction layer, and the emotional layer [71]. The lower-level layer constitutes the foundation for the upper levels. The focus in this paper is on the middle-level layer, since a UX evaluation of action and intention recognition primarily focuses on the interaction between the human and robot. However, it is important to make sure early in the development cycle that the robot’s ecology is appropriate. The ecological layer concerns the conceptual design and whether or not it fits the ecology in which the robot is going to act, i.e., its complete surrounding such as other systems, information structures, instruments, and users [71]. A bad conceptual idea of the robot in the sketching phase can, without high costs, be completely changed, but if it is discovered in a late prototypical stage that the robot concept is flawed, it can be impossible or at least highly expensive to solve the problem. One way to address this is by doing an informal evaluation of the robot, sometimes also denoted ‘quick and dirty’ evaluation [139] or quasi-empirical UX evaluation [71]. Conducting an informal UX evaluation could be considered depending on the current status of the robot project. The informal evaluation could be performed with rather simple means and efforts, e.g., creating basic sketches of alternative appearances of a new social robot, raising critical questions in the planning of introducing an existing robot to another user group or applying it in a new context of use. Using this way of working, it is possible to receive some hints of potential possibilities and problems that can be used to guide upcoming decisions on further efforts in the robot project. When doing informal UX evaluations, rather basic and simpler presentation materials could be used such as sketches, drawings of envisioned interaction flows, storyboards, and scenario descriptions, which are used by the investigators as input during discussions with end-users, which could be carried out individually or in groups [71,92,93]. Informal evaluation should not be used when there is a need for a rigorous UX evaluation [71], i.e., a comprehensive UX evaluation that combines an analytical and larger empirical UX evaluation, but is suitable under circumstances when the time and resources are not available to do a thorough investigation, because at least it is possible to receive quick feedback from end-users on initial concepts and ideas.

#### 4.2.2. Characterization of Analytical and Empirical UX Evaluation

From a general point of view, UX evaluation on the interaction and emotional level can be either analytical or empirical [137]. *Analytical UX evaluations* differ from empirical evaluations in that they do not involve user participants [140]. Instead, HRI or UX experts inspect or walk through aspects of the intended interaction with the aim to predict the future user experience of the human-robot interaction [5,141,142,143,144]. Weiss et al. [144] have made a modified version of heuristic evaluation for HRI, although not focusing on action and intention recognition *per se*. This makes it possible to identify, e.g., potential problem areas, which for instance can guide decisions on focus areas for further investigations or design efforts within an ongoing project. The outcome of analytical evaluations are often a list of problems and ideas on how to address them [5,93,141,142,143,144]. A way to evaluate HRI predictively is to use theories or models of human activity. In the case of evaluating action and intention recognition, the modified seven stages of action model (see Section 3.2 and depicted in Figure 3) can be used as a way to systematically analyze the interaction to be within the frame of the defined goals, end-users, tasks and context.

*Empirical UX evaluations* can be either UX testing or field studies [5,71,145]. UX testing is a more rigorous way of evaluating specific aspects of the interaction between human and robot, e.g., evaluating the degree of correctness regarding prediction of what a robot will do next, assessing the experience of certainty when interpreting a robot’s intention signifiers after a first short-term interaction, or identifying problems on how to respond to the robot’s actions. This kind of UX testing is often conducted in a laboratory setting or using a demonstrator set-up in which the variables and context can be controlled [71,92,93]. It should be highlighted that many human-centered researchers fail to distinguish between “UX testing” and “user research” in the form of contrived experiments performed in laboratory settings that are usually derived from experimental psychology. Due to the fact that they are rather similar at a first glance, the confusion is understandable because both approaches are empirical by nature: using sampling techniques of participants, centering on observations on actual behaviors performed in a laboratory setting, and collecting and analyzing quantitative and qualitative data. Taking a deeper look, however, reveals significant differences in other respects (see [64,71,93] for further details). The differences are manifested in, e.g., the regular set-up for UX testing, in which the user participants interact with the robot based on specific scenarios, within which certain tasks are performed with lesser control by the investigator than in an experimental setup. This means that the user participants typically are encouraged to explore by themselves how to interact with the robot to achieve the tasks at hand presented in the scenario. Pre-test, during, and post-test inquiries can be included in the forms of questionnaires and/or interviews [92,93]. The collected primary data is often quantitative to be used to compare the obtained results to specified UX goals and UX measures and for benchmarking tasks with other robot platforms or verifying the obtained effects of improvements of the robot design. Accordingly, qualitative data is usually also collected, having the purpose of facilitating and gaining a deeper understanding of the interpretation and comprehension of the obtained quantitative results [64,65,66,67].

Moreover, field studies are not like UX testing, being conducted in a natural environment in which the investigators have less possibilities to control various factors and conditions in the context of use [145,146,147,148]. Although the lack of control in field studies might seem problematic, we want to emphasize that in the long run, the majority of robots are expected to be situated in a natural environment, which could either be in a highly specified or in generic contexts. Hence, this implies that, eventually, it is in this kind of ecological or natural environment that the action and intention recognition of humans interacting with robots should be implemented and completely functioning. As an HRI researcher, one needs to be aware of the fact that it is not always possible to transfer results from a controlled situation or context of use to a natural and, thus, more messy and unconstrained environment [147,149]. However, there exist results which indicate that UX testing in a laboratory setting is able to identify, to a large degree, the same UX problems as those revealed in a field study [150]. Thus, to be able conclude and decide upon the actual AIR UX qualities, it is necessary to conduct field studies. In field studies, the primary collected data is often qualitative, collected using, e.g., various kinds of observations, interviews, and diaries, because the possibilities to provide measures with high accuracy is limited due to the uncontrolled and messy environment [5,64,151]. Nevertheless, some quantitative data can still be gathered, e.g., via questionnaires like AttrakDiff [152] and SUS [153], using behavioral protocols for counting the frequency of positive and negative facial expressions as well as verbal utterances, measuring the time on tasks, or measuring the number of errors that are made, which altogether could add complementary perspectives and nuances to what end-users are actually saying and doing in the wild [5,71,76].

### 4.3. Phase 3: Plan and Conduct the UX Evaluation

Depending on the purpose of the UX evaluation, the investigators can decide to initially perform an analytic UX evaluation, and the obtained results could be handled before doing an empirical UX evaluation. It is preferable to conduct the analytical one before the empirical one, if the empirical UX evaluation should be rigorous [64,71,92,93].

#### 4.3.1. Analytical UX Evaluation of Action and Intention Recognition

To identify potential problems regarding human recognition of a robot’s actions and intentions it is possible to use a modified version of the cognitive walkthrough [5,64,141,154], but instead of using its original questions, we suggest to use the modified seven stages of actions (see Section 3.2, Figure 3 [91,105]). This means that one or more investigator(s), preferably with some expertise in HCI, UX, and HRI, systematically walk(s) through a scenario step by step and answer some predefined questions. Before the analytical UX evaluation can occur, the following aspects need to be prepared and available:

- Knowledge and experience of the end-user group, e.g., if the situation is well-known and if the task is familiar to the end-users.
- A scenario that portrays the context, the goal of the activity, and includes representative tasks.
- The robot that is either functioning or is complemented with a description of the relevant interactive aspects. A Wizard of Oz set-up or a film of the robot in action, although not recorded with an evaluative purpose, can also be used.

During the analysis, the investigator(s) walk(s) through all specified tasks in the scenario answering the following six questions. Depending on the scenario, the walkthrough begins either with question 1 (data-driven activity, i.e., the activity is triggered by the robot in relation to the context) or question 4 (goal-driven activity, i.e., the activity starts with a user wanting to achieve something).

1. Is it easy to perceive the intention of the robot to perform an action, i.e., perceive the robot’s status in its context?
2. Is it easy to predict what sequence of actions that will take place, i.e., interpret the robot’s status in its context?
3. Is it easy to assess the robot’s actions in the relation a person’s goals, i.e., evaluate the interpretation in relation the goals?
4. Is it easy to determine if any measures need to be taken in relation to the robot in order to reach the goals, i.e., forming intention to act?
5. Is it easy to specify proper sequence of actions in relation the robot, i.e., understand and determine what is possible to do in order to reach the goal?
6. Is it easy to perform the chosen sequence of actions in relation to the robot, i.e., execute the sequence of actions?

All identified problems from the walkthrough should be described and explained. Then, the investigators should assess the severity of the identified problems (see the analyzing phase described in Section 4.5 for further guidance).

#### 4.3.2. Empirical UX Evaluation of Action and Intention Recognition

Based on the UX goals (formulated in phase 1), a relevant type of UX evaluation is chosen (phase 2) and then what kinds of data gathering techniques that should be used, e.g., observation, video recordings, interviews, and/or questionnaires [64]. The next step is to characterize the end-user profile of the user participants, both in terms of typical and diverging characteristics. These characteristics have to be adequately defined and quantified [71,92,93]. The joint attribute could be end-users of both sexes that are more than 75 years old, who are living alone at home, and have no prior experience of socially assistive robots. The diverging aspect could be positive and negative attitudes towards social robots in general. From the initial joint group of elderly end-users, two subgroups could then be created, which in this particular case would enable the investigators to make comparisons between the two sub-groups, constructed on diverging initial positive or negative feelings towards social robots. In so doing, the investigators would be able to draw conclusions with regard to the earlier defined UX and AIRattr goals in the first phase of ANEMONE (see Figure 4 and Table 1). From a practitioner perspective, the major output from the UX evaluation could be to be better informed about to what extent the current socially assistive robot’s physical appearance and its behaviors, i.e., ways of interacting and communicating with the end-users, sufficiently fulfil the defined UX and AIRattr goals. If the defined UX and AIRattr goals of the robot’s appearance and aspects of the interaction quality are not yet partly or completely fulfilled, the obtained outcome offers valuable hints of what the problems are and tentative ways on how to elucidate or reduce them. It should be mentioned that the outcome is not assumed to infer generalizable claims. This means that in the context of commercial robot development, the number of user participants does not have to be high, because the involvement of only five user participants in each subgroup can provide sufficient information and insights to perform relevant changes of the robot design [71,93,141,155,156], especially if the design changes are not extensive and fundamental ones, but rather of an incremental sort. However, in the case of major and expensive changes, a larger number of user participants or conducting several UX evaluation rounds are recommended, which, from the long term perspective, might be a good investment for the company.

Before the recruited user participants begin the actual UX evaluation, some ethical concerns need to be raised [71,157,158]. It should be emphasized once again that UX evaluation differs significantly from experiments [64,93]. In UX evaluation, the user participants are not subjects that are manipulated or tested; it is the interaction quality and problems that occur during the interaction that is the main phenomenon of study. Hence, the user participants are tentative end-users who help the investigators to test the interaction between robots and humans for pragmatic and hedonic qualities, but the user participants in an UX testing as such are not tested. In so doing, the end-users become user participants or sometimes partners in the process of evaluating the robots [71,157,158]. However, when collecting data from humans, there are legal and ethical responsibilities and obligations to consider, although there is a minor risk for user participants to be harmed during UX evaluation [71,157,158]. These obligations include formulating and using an informed consent form, which is a document provided to the user participants that covers their rights, the aim and the nature of the UX evaluation they are asked to participate in, and contact information of the investigators. The form also serves as a legal protection for the investigator(s) and their organization and should be signed by the user participants. The informed consent form must declare that participation is completely voluntary, and the user participants need to be properly aware of the purpose and aim of the evaluation, the procedure, the intended benefits of their participation, and any discomfort that may be induced during the evaluation. In most countries the informed consent form, together with other details on the UX evaluation, have to be applied to an institutional review board (IRB), which approves the evaluation. We want to highlight the need to carefully consider safety issues if user participants should interact with a robot prototype or demonstrator that is in the development phase. Moreover, the user participants should be informed about their right to end their participation at any time during the evaluation without any negative effects. The user participants should be informed how long the evaluation would take, what kind of data that is collected, how the data will be used and stored and by whom and for how long, any limits of confidentiality, and how to get in touch with the investigators if any questions or concerns should arise (for further details, see [71,158]).

It should be emphasized that the tasks, often presented in scenario(s) that the user participants are going to perform during the UX evaluation session with the actual robot need(s) to be thoroughly planned and prepared so they are (i) connected to the earlier defined UX and AIRattr goals and (ii) relevant for the intended end-users to conduct. The tasks that the user participants should carry out could vary from being a short initial training session that aims to familiarize the participants with the particular setting and the actual robot to the major performing part of the UX testing that needs to be carefully prepared [92,93]. It is recommended to run a pilot test, i.e., a kind of pre-test or dry run, if the UX testing is rigorous and involves many user participants, including the whole UX evaluation procedure as well as double checking that the equipment intended to collect data functions properly. A commonly used approach to make the UX testing more natural for the user participants is that the tasks that the participants should achieve are presented in a scenario-based form. The scenarios should contain some short and explicit stories, which provide some support to the user participants involving certain goals they have to achieve when interacting with the robot and necessary level of information to carry out the tasks at hand [71,92,93,159]. There exist several ways to present the scenarios to the user participants, e.g., written instructions on paper, a short video-clip, verbal instructions by the investigator, using a role play, or by the robot itself. The last suggestion is an appropriate way to do it, especially in the case where the robot is intended to be a social companion; it could be convenient if the robot provides the scenario to the user participant and then guides the user participant through the whole session. Depending on the developmental stage of the robot, it could be the case where a real interaction between the user participants and the robot is not yet possible, then the Wizard of Oz (WoZ) technique could be used as an alternative way of acting [5,100,160]. The WoZ technique is realized when a human from the investigator team plays the robot interaction parts in a puppet manner, which allows evaluation of the envisioned interaction flow between human and robot at an early stage of robot development when certain features have not yet been implemented in the robot.

**Figure 5:**
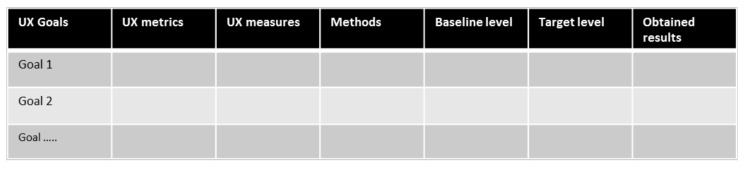
Evaluation matrix (adapted from [66,67,71]).

It should be possible to measure the UX and the AIR-specific goals and aspects with objective as well as subjective measurements [64,138,161]. This is particularly important in UX testing in a laboratory setting, but it can also be advantageous in a field study. However, in the field, where the variables cannot be fully controlled, the measures should be carefully treated. There is a wide range of aspects that it is possible to quantify. UX measures are linked to the UX goals, and UX measures are the general characteristics of the UX issue to be measured, e.g., learnability, initial performance, initial impression, long-term impression, ease of interpreting robot’s actions at first encounter, ease of interpreting the robot’s actions after five trials, etc. [71]. UX metrics describe the kind of value to be obtained, being quantitative or qualitative in nature, such as time on task, amount of errors, subjective rating scores on a post-test questionnaire after the first impression as well as after five trials [71,102]. It can be several UX metrics for a given UX measure. Objective measures can be time, degree of completion, and correctness [64,161]. Subjective measures can be grading of experiences (e.g., perceived safety, experienced trust, smoothness of task-switching) or preferences between different alternatives [64,161]. There should also be reference values for the measure, such as level of acceptance (baseline level) and desirable target level [64,65,66,67,68,71]. These levels could be portrayed in a UX evaluation matrix [71,102], which is illustrated in Figure 5. Putting the relevant aspects for the UX evaluation into a matrix enables a better overview of the evaluation outcome, which we highly recommend.

**Example**

*Pragmatic quality—objective UX measure*: Number of errors is the UX metrics, and the UX measure is error performance, with the reference points for the latter is that one error per user and task is accepted (baseline) and no error is desirable (target).

*Hedonic quality—subjective UX measure*: The UX measure is user opinion of confidence in communicating with the robot and the grading of confidence in communicating with the robot on a scale from 1–10 where 1 is the lowest and 10 is the highest UX metrics (quantitative form of subjective ratings). The UX methods are questionnaires and interview, with an accepted average baseline level on 6 and desirable target level on 8 for the questionnaires.

*Objective AIR measure*: Percentage of visitors perceiving that the robot turns its face and body towards them in a crowded entrance of a shopping mall is the UX measure, the UX metrics are the percentage of perceiving the robot correctly, and the method used is observation, where 50% is accepted and 80% is desirable.

*Subjective AIR measure*: Grading of certainty of interpretation of what the turning of the robot means is the UX measure, and the UX metrics is done on a Likert scale from 1–5 where 1 is the lowest and 5 is the highest UX metrics (ratings). The UX methods are questionnaires combined with observation (interpretation of the investigator) and interview, with an accepted average baseline level on 4 and desirable target level on 5 for the questionnaires, which are combined with the analysis of the qualitative data.

In addition to the quantitative UX metrics and UX measures, it is advantageous to collect qualitative data in order to gain a deeper understanding of why the interaction unfolds as it does, why problems occur, and why the UX measurements end up as they do. Observations (combined with video recordings) of the human-robot interaction from multiple angles and a post-test interview could be valuable. We recommend the use of video-recordings to complement the observation, because the recordings could be valuable in the analysis phase as well as to demonstrate for other parties what happened during the interaction with the robot. Inspiration for creating interview and questionnaire questions can be found in the modified version of the seven stages of action model already presented (see Section 3.2), although more open questions beyond that can also be valuable.

**Example**

- When you see the robot located near the door, what do you think that means? Why?
- When the robot moves towards you, what do you think that means? Why?
- Your reaction to the robot’s gestures was to move backwards, can you tell us the reason behind that reaction?

### 4.4. Phase 4: Analysis of Collected Data and Identifying UX Problems

#### 4.4.1. Strategies for Analysis of the Collected Data and Identifying Problems

When the UX evaluation session, which can include several scenarios, rounds, and several kinds of user participants, has been carried out, the collected quantitative as well as qualitative data should be brought together and then analyzed, with a focus on identifying UX problems in general, and specifically UX problems of action and intention recognition that need to be handled. As already described in Section 3.3, UX comprises of both hedonic and pragmatic qualities, the latter commonly known as usability [129,162]. ”A usability problem is an aspect of the system and/or a demand on the user which makes it unpleasant, inefficient, onerous or impossible for the user to achieve their goals in typical usage situations” [162] (p. 254). To be certain that an identified problem is authentic and that there is a need to do changes, *triangulation* is a preferable way to obtain more reliable findings.

Triangulation means that multiple data sources are used to compare and contrast the data in order to gain a deeper understanding of obtained findings [64,92,93,151,163]. Several findings that are pointing in the same direction imply that there is an identified UX problem that needs to be considered. A UX evaluation can comprise of a problem list generated from an analytical UX evaluation (see Section 4.3.1 [141,143,154]) and quantitative metrics and measures, e.g., time to perform a task and amount of errors made. Qualitative aspects also provide some insights, e.g., interview answers regarding perceived efficiency or questionnaires that use Likert scales to assess the trustworthiness of interacting with the robot, which could be obtained from empirical UX testing (see Section 4.3.2). A tentative way of working with the collected data goes as follows. If there is a listed potential UX problem that can result in interaction inefficiency, the investigator could calculate if the measured mean value of the time to execute a certain task is below the defined acceptance baseline level. If that is the case and the user participants frequently made various kinds of verbal utterances and facial expressions describing and displaying the interaction with the robot as frustrating, then there is strong evidence of an actual difficulty that can be assumed to cause real-world struggles and the outcome of negative UX. If it is the case that one of those findings described above is pointing in another direction, e.g., the time to execute the task reached the defined desirable target level, then it is recommended to further and more deeply investigate the tentative UX problem, before taking actions to address or solve it.

#### 4.4.2. Analysis and UX Problem Identification of Action and Intention Recognition

To analyze, describe, and report the nature of the UX problems and understand the emergence of them [71,162,163,164], the modified seven stages of action model can assist in classifying and structuring the identified UX problems. In so doing, the seven steps could be used to identify *when* problems of action and intention recognition occur between the human and the robot. Several loops in the action cycle might need to be performed in order to identify different kinds of problems that occurred during the scenario and tasks used.

As pointed out by Law and Sun [88], Rogers [76], and Halverson [119], AT brings a structure for analyzing the qualitative data. AT enables the grouping of collected data into basic units of analysis in the form of activities, and the AT concepts function as a filter, ‘pair of glasses’, as Halverson [119] puts it, or focal points to guide the analysis to identify the problems. Furthermore, AT’s hierarchical structure of activity (see Section 3.1.2, Figure 1) can be used to zoom in and out, meaning that the action-goal level that is in focus can be viewed rather from a macro perspective, i.e., activity-motive level, or a micro level, i.e., operation-condition level [76,119].

**Figure 6:**
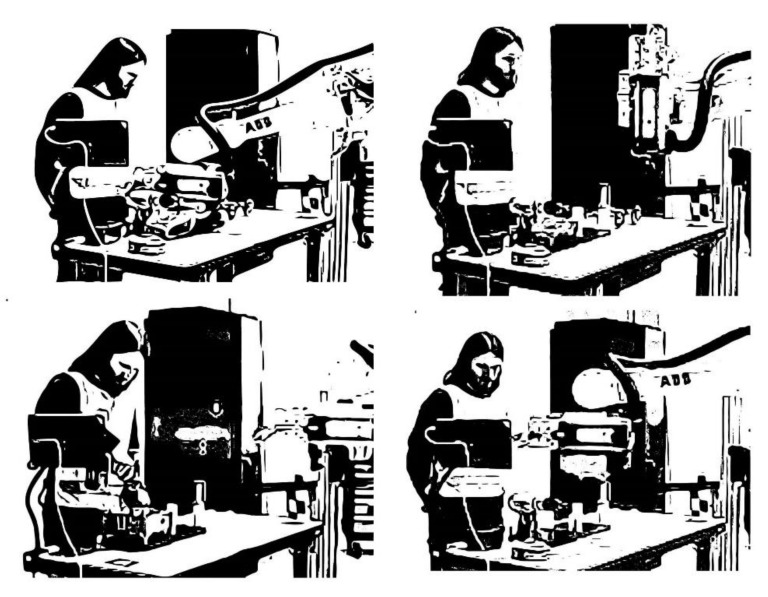
Illustrations of human-robot collaboration of assembly in manufacturing.

Problems that occur in the interaction between human and robot can be related to circumstances in the context, which means that contextual aspects need to be included in the analysis. In that sense, AT can support a better understanding of the context under which UX unfolds [76,80,81,82,83,84,85,86,88,119], because it is tricky as well as unsuitable to understand an activity and to identify UX problems without considering the context, which is the main foundation of AT. In order to better grasp the different essential parts of the evaluation context, relevant for the evaluation at hand, aspects from Engeström’s triangle/ASM [117], the activity checklist [105], and the eight-step model of activities [121,122] are adapted and modified in Table 2 and Table 3 (see Section 3.1.4). These tables provide guidance in the form of focal points to consider during the UX analysis of the human-robot interaction situated within the context in the area of interest to identify UX problems and tentatively gain a better understanding of *why* they occurred. In order to make the content in the tables more comprehendible, they are portrayed from a real UX evaluation situation of a human-robot collaboration demonstrator of assembly in manufacturing that was carried out within a project that we participated in (Figure 6).

Other relevant issues to consider during the analysis and in identifying UX problems in the interaction between the human and the robot are to identify gulfs of execution or gulfs of evaluation in the action cycle of the modified seven stages of action model (see Section 3.2), breakdowns [88], or contradictions [109] between various components in the ASM or between different ASMs (see Section 3.1.3).

### 4.5. Phase 5: Organizing the Identified UX Problems in Scope and Severity

The final phase in performing an UX evaluation, either an analytical or empirical one, is to arrange the identified UX problems into scope and severity [64,92,93]. The *scope* of an identified UX problem can be either global or local, in which the scope is global when the UX problem covers the interaction with the robot as a whole, and the scope is local when the UX problem occurs only in (a) certain task(s) [64,92,93]. A tentative example of a global problem is when the provided means to communicate with the robot is by gesturing only. If the gesturing mode does not function appropriately, then it is considered a global problem because it has a negative impact on all interactions between humans and the robot, resulting in negative or bad UX. A tentative example of a local problem is when the user is unable to comprehend a particular signal from the robot, although the interaction flow as a whole runs smoothly. It should be pointed out that global problems usually are viewed as more serious than local problems [64,92,93]. A global problem could also go beyond a certain robot, being part of a whole family of robot products/platforms, if the problematic aspect is integrated in several kinds of robots. Nevertheless, it is not always the case that global problems are more severe than local problems. A local problem could be very critical, e.g., if a user cannot interpret a specific signal from the robot, although it happens only now and then, but it should be considered if this particular signal stands for a severe security risk for collision when collaborating with the robot in a shared workspace. In that case, the local problem is of high gravity.

The identified UX problems should, beyond the terms of scope, also be properly analyzed in terms of *severity*. The severity of an identified UX problem offers a key factor for making relevant decisions about which kinds of re-design actions should be prioritized in the interactive UX design lifecycle process [71], being aligned with asking how resource-demanding the re-design actions are in terms of, e.g., setting/context of use, time, money, competence, and skills, as well as the availability of these resources [92,93] (see Section 3.3). The common way to organize the degrees of severity is ranging from high to low (sometimes the degrees consist of a numbered scale), where the highest degrees include, e.g., severe safety risks and UX problems of any kind that hinder or delay the completion of central tasks in the interaction between a human and a robot. Lower degrees of severity are identified UX problems that have a minor negative effect on the human-robot interaction, as in situations when it is easy for the user to find an effortless workaround while performing the central task(s), if the UX problems concern tasks that are not critical and seldom occur, or if the UX problem is of more cosmetic nature [92,93,148]. Furthermore, other relevant aspects that should be carefully considered when stating the degree of severity are, for instance, the number of end-users that is expected to be affected by the identified problems, e.g., related end-users with certain characteristics, if the UX problems cause breakdowns and interruptions, or with what frequency the problem occurs. Thereafter changes in the design or interaction flow between the human user and the robot should be suggested for how to solve or reduce the negative effects of the prioritized UX problems [92,93], which is part of the iterative nature of the UX design lifecycle process [71].

We touched upon contradictions in the earlier phase, and in ASM [117] (see Section 3.1.3), contradictions are viewed as sources of development, because humans try to handle these contradictions in practice, based on their own motives and activities. Therefore, it is important to consider identified contradictions from several perspectives, shifting focus from the actions of the individual to zooming out to the broader activity context and then zooming in again. Thus, these contradictions are viewed as the driving force for development and should be further investigated and analyzed before any re-design is decided upon. As pointed out by Bødker [118,137], practice is constantly evolving, it never stands still, changes of the mediating artifact, i.e., the robot, and other tools/instruments alter work practice, and the changes in work practice reshape the robot and the tools/instruments used [80,81,118,137].

Visualization is another issue to consider when portraying and presenting the identified UX problems, and their scope and severity. By making visualizations of the UX problems, it can be easier to communicate and discuss the UX problems with robot developers and other representatives. Law and Sun [88] have developed specific graphical notations to indicate, e.g., a breakdown within Engeström’s triangle [117]. Halverson [119] points out that these visualizations provide a major strength of AT. Other ways to visualize UX problems is to use short video clips from empirical UX testings to show what happened and provide tentative explanations of why it occurred.

The identified UX problems could then be re-design in the robot itself, or adjustments could be made in the other tools and instruments used, e.g., the assembly instructions provided on the tablet for a robot used in manufacturing assembly. After the re-design is implemented, a follow-up empirical UX evaluation round could be performed, to reach the UX goals that are not fulfilled yet. Sometimes, further investigations need to be performed for a certain aspect of action and intention recognition, and for that particular situation, more detailed data can be collected from eye-tracking equipment. On other occasions, the tasks may need to be reformulated, because they are not well aligned within the context of use and/or the current work practices.

## 5. Discussion

In this paper, a systematic methodological approach called ANEMONE for evaluating humans’ possibilities to correctly and with certainty perceive, understand, and predict the actions and intentions of a robot is presented. The aim is to provide guidance on how to carry out UX evaluation and facilitate an understanding of why something works or not, by identifying UX problems. The ANEMONE approach uses AT [76,80,81,82,83,84,85,86,88,103,104] as a theoretical foundation, which provides a lens through which the phenomenon of activity is portrayed in the context of HRI. The building blocks of the approach are UX evaluation methodology [71] and a modification of the seven stages of action model [91,105]. All three parts are well-established in HCI separately, and by combining them, a solid methodological and theoretical foundation for UX evaluation of action and intention recognition in human-robot interaction is obtained.

As portrayed in Section 2, the method development process of ANEMONE has not occurred ad hoc or through a strawman approach, which seems a rather common approach when we made some inquiries about how to properly develop an evaluation method when this work was initiated. Our intention to use Blandford and Greene’s [94] approach was two-fold. On the one hand, their method development process provided a proper support for us to reflect on several relevant aspects, which otherwise may have been excluded. On the other hand, following their method development process [94] offers ANEMONE trustworthiness that the method has credibility, given that the authors have extensive experience and skills in developing various evaluation methods over the years, several of which have been considered rather successful [76,94]. Upon reflection on how well Blandford and Greene’s [94] approach has supported the development of the methodology of ANEMONE, we realize that we have not used their approach as straightforward as it is described in Section 2. In hindsight, it is obvious that we have not followed the stepwise order, instead, we have used it more iteratively. During the initial design and development process, we had rather vague ideas about what our methodology should focus on in more detail as well as its focus. The initial workshops focused a lot on robot intentionality, and we were often involved in interesting discussions from a cognitive science perspective, but which were not so fruitful for the creation of ANEMONE. One reason for not advancing forward in the very beginning was the lack of deep understanding of the underlying cognitive mechanisms of action and intention recognition between humans and robots, as well as the more cognitive science related and much debated issue whether robots could be considered to have intrinsic intentionality or not. After a while, we realized that we needed to take a different direction than the more embodied cognitive science-oriented perspective. We therefore focused on designing and developing a UX evaluation method, and during another workshop, this direction turned out to be more promising, because the intended benefits and usefulness of the envisioned ANEMONE approach method could reach a larger audience preferably than cognitive science researchers, as well as there being a lack of similar approaches. When we reached that decision, it was easier to proceed according to Blandford and Greene’s approach. However, we spent some time before we reached a shared understanding of the initial phases of their evaluation method development, and we went back and forth between the second and third phases, which means that the method-in-concept and the method-in-tool were not yet clearly formalized for ANEMONE. We also participated in HRI research projects that provided some input on how evaluation of action and intention recognition from a UX perspective could be performed. The main contributions of Blandford and Greene’s approach, in our view, are the following ones. Firstly, it provides several aspects that need to be considered when developing a method/methodology, which otherwise can be neglected. The many issues raised in the second and third phases are necessary to consider and to decide to what extent they should be dealt with as well as in what ways. The issues listed in the second and third phases are significant aspects that should be considered to a larger extent in method/methodology development in general. Second, the structure of the five phases supported our own understanding of what we were doing and when and served as a discussion tool during the design and development process. Thirdly, Blandford and Greene’s suggestion to use existing methods and combine them in novel ways inspired us, and that is the major underlying reason for combining AT, Norman’s seven stages of action model, and UX evaluation methodology. The tricky part was how and in what ways they should be combined and how to transfer them to an HRI context. We did not find any support for this challenge in their approach [94], which is the focus in the fourth phase, which they actually spelled out. In this particular phase, there is room for further elaboration. Although they cannot provide any stepwise recommendations how to proceed, some more support than the PRET A Rapporter framework would be appropriate to develop further. The fifth and final phase is the testing phase, and we have not yet systematically tested the whole ANEMONE approach in practice yet, i.e., method-in-action, although the workshops have provided valuable input to consider. Many issues have been raised concerning the nature of UX evaluation methodology. Therefore, we have included many references to initial and more classical publications within the HCI and UX fields to offer credibility to the ANEMONE approach. We also have extensive teaching experience of lecturing and supervising students on usability and UX evaluation methodology, which we found to be useful in this work. To summarize, the pros of using Blandford and Greene’s approach are greater than the cons.

Furthermore, a relatively often discussed issue initially during the development process of ANEMONE was the nature and rigor of UX evaluation methods. We have experienced that UX evaluation as such is sometimes considered less scientific and less rigorous than psychological experiments that currently seem to be the most frequently used method to evaluate how humans interact with robots within the HRI field [7,8,68]. We emphasize that this way of arguing is like comparing apples and oranges, because their aims and purposes differ significantly. Instead, our ambition is that ANEMONE portrays a valid and credible approach for identifying UX problems that hinder or break the envisioned fluent and smooth interaction between humans and robots, in which action and intention recognition is the means for this intuitive interaction to emerge. We do not exclude that employing an experimental approach could be a viable approach for further investigation of a specific identified aspect of a UX problem in more detail. This reflection on methods used in HRI is well-aligned with Dautenhahn’s [68] worry that experimental psychology to a large extent has been adopted as the proper standard of conducting empirical work in HRI. She addresses the need for new evaluation approaches for human interactions with robots, as we presented in the Introduction [68] (see Section 1.2), which she has also emphasized in her earlier publications [165,166]. We have noticed that UX aspects are often excluded in HRI studies, and instead, robot-related aspects are usually favored. In other words, the main focus of study is on investigating how the robot itself affects the users, i.e., one-way communication, which is not the same as studying the two-way interactions between humans and robots [25]. Consequently, by adding a UX perspective, the outcome could be even more valuable, and the acknowledgment of the complexity of UX could contribute to more credible and valuable findings and results. We advocate that increasing the recognition of the many facts of UX is likely to make the contributions even more valuable because more insights could be derived for both practice and theory.

The fields of UX and HRI have both been questioned in regard to the lack of proper theoretical foundations. In the UX field, this issue has been raised several times (e.g., [88,89,90]), and the same holds for HRI [1,165,166]. By using AT as a theoretical lens in ANEMONE, we hope that the theoretical perspective in our work will be beneficial to both the UX and HRI fields.

### 5.1. ANEMONE’s Alignment with AT: Pros and Cons

According to AT, human activity can only be understood and described in its context [80,81,82,83,84,85,86,88,116,118]. Therefore, the presented methodological approach of ANEMONE emphasizes the necessity of doing thorough preparations in setting the scene by defining the context and the use of scenarios as drivers of analytical as well as empirical evaluations. Four of the central principles are integrated in the methodological approach: hierarchical structure of activity, object-orientedness, tool mediation, and internalization-externalization. The fifth central principle, development, has received less attention, but the obtained UX findings in the form of UX problems, particularly contradictions and breakdowns, obtained from the UX evaluation provide valuable insights for the future design and development of robot-mediated interaction. As emphasized in AT, there is always a historical perspective on tool-mediation, and to gain a deeper understanding of any phenomenon, it is necessary to know how the robot has developed into its current design and function. This principle is related to the iterative “UX wheel”, which consists of the four key elements of UX activities: analyze, design, implement, and evaluate [71] (see Section 3.3). In this paper, we have mainly focused on evaluation activity, i.e., the different methods and techniques that can be used to investigate and analyze to what extent the proposed robot-mediated interaction meets the users’ needs, requirements, and expectations of action and intention recognition. The whole UX wheel corresponds to an iterative UX design lifecycle process that is accompanied by the identified and characterized UX goals [64,65,71].

AT provides a *hierarchical structure of activity*, with the activity levels activity-motive, action-goal, and operations-conditions (see Figure 1) [84,85,86,104]. As the focus of the methodological approach is on humans’ possibilities of perceiving the actions and intentions of robots, the center is on the action and goal level where Norman’s modified seven stages of action model [91,105] provides a more specific structure for an evaluation. Nonetheless, by viewing what is happening on an action-goal level in a hierarchy makes it possible to zoom out, i.e., put the action-goal aspects in a larger setting, and zoom in, i.e., breaking the aspects of concern down into details. Changing the level of details during analysis of findings can facilitate an increased understanding of what is happening and why the interaction unfolded as it did. As pointed out by Raeithel and Velichkovsky [167], the *hierarchical structure of activity* could also be used as a guide for proper change of focus and choice of methods to be used in further evaluation. On the one hand, they suggest that ethnography could be a viable approach for studying and evaluating historical and sociocultural development from the activity/motive level, zooming out from the action level. On the other hand, they suggest that tracking gaze-following behavior in joint attention experiments could be a viable approach for investigating microgenetic and conditioned factors, zooming in from the action level. 

*Object-orientedness* emphasizes objects as drivers behind human activities, where an object can be mental (e.g., an ideal) or physical [86,116]. By using a modified version of Norman’s seven stages of action model [91,105], the driving forces are present in the approach where the action begins as either mentally, as a goal that needs to be fulfilled, or physically, with perceiving a state in the context that is not in line with the desired state. Goals and desired states are verbalized in the methodological approach as goals, e.g., user goal and UX goals.

In the terminology of AT [86,118,137], the robot is an artifact that *mediates* the human’s actions in order to fulfill the user’s motives (activity in AT). As a mediating artifact the value is not in the robot as such, instead, the value lies in how “good it assists” in obtaining the goals (motives) of the actions. Thus, it is essential to specify what the goals are, as stressed in the proposed methodological approach. It is in relation to the goals that it is possible to assess if the aim and intended value is created or obstructed.

*Internalization-externalization* concerns the reciprocal actions between the explicit (e.g., physical movements, utterings) and the implicit (e.g., thoughts, intentions) [80,81,82,83,84,85,86,116]. This is the core of UX where the implicit, i.e., the inner experiences and feelings of the human, meets the explicit, i.e., the interaction between the human and the digital artifact [78,79,80,81]. Thus, by using UX evaluation methodology as a fundament in the methodological approach of ANEMONE, the principle of internalization-externalization becomes naturally integrated. The modified seven stages of action model also embraces this principle by comprising physically doing as well as mentally experiencing as imperative parts of conducting actions, being two sides of the same coin.

However, various kinds of criticism have also been formulated against the AT approach and the activity system model (ASM). As pointed out by Susi [116], one of the major criticisms that AT has encountered is the problem of explaining a concept through the very same concept, i.e., explaining activity through activity, since the concept of activity is used both as an explanatory principle and as a subject of study. Some of the common raised criticisms are that the success of using and obtaining results from AT research in HCI largely relies on the analyst’s skills of handling and interpreting the collected data in a coherent way when relating it to the concepts of the AT framework [76,119]. There is no obvious methodological description for data collection or data analysis and particularly limited guidance for determining the different levels of activity in a particular context where they occur [76]. It is therefore argued that it takes time to obtain an acceptable level of understanding and competence in using AT appropriately. Simplifications of the AT framework or ASM [117] could then result in less utility of the obtained results, given the unfamiliarity with the historical roots, basic principles, and context of AT [76,119]. Despite the above raised criticisms, AT is one of the most influential approaches used in HCI [76,84,85,86,103]. Rogers [76] addresses that the ASM has been used to analyze varying kinds of work settings, particularly when there are problems with current or newly implemented technology, where the ASM makes it possible to identify both micro and macro level issues. As more robots enter the landscapes of work, e.g., manufacturing, we foresee an increased use of AT and ANEMONE in workplace analyses. The structure and processes gained from UX evaluation and the modified seven stages of action model [91,105] provide some support in doing the analysis, and the structure and procedure of ANEMONE tries to overcome these addressed limitations of AT.

### 5.2. Pros and Cons of the ANEMONE Methodology

The major pros with the ANEMONE approach are the identified need of a UX evaluation methodology that is based on a solid theoretical foundation for considering action and intention recognition between humans and robots. Furthermore, ANEMONE is based on theories, models, and methodologies that separately have been very successful and influential in the HCI and/or UX fields, and hopefully, their combination will also be beneficial for the HRI field. By following Blandford and Greene’s evaluation method development approach, this way of working hopefully provides some credibility to ANEMONE, in particular, the *construct validity* should be considered very high. The ANEMONE approach is a methodology, i.e., it is a bunch of several UX evaluation methods, being analytical as well as empirical. Therefore, several method-in-tools exist within the ANEMONE approach. The theoretical foundation in AT can, besides being considered a strength, also be considered a weakness, because the AT terminology may at a first glance be rather challenging to grasp and the analysis requires a skilled AT analyst, although we have provided some guidance in Section 4.4, especially for the qualitative data analysis phase [76,119]. The level of detail in ANEMONE can also be considered as both a pro and con, which refers to *structure*. On the one hand, depending on the reader’s and investigator’s field of expertise and prior experience, some aspects are perhaps described in too much detail. On the other hand, there are certainly issues that could have been presented and discussed in more detail than currently done.

One of the major cons with the ANEMONE approach relates to the lack of systematic testing of the methodology in practice, i.e., the method-in-action perspective. Blandford and Greene [94] mention that evaluation method development usually takes time, and they point out that many well-known methods, such as cognitive walkthrough [142], have been further developed over more than a decade. As pointed out by Blandford and Greene [94], testing is of major importance, and it is different to follow a method in theory and handle it properly in practice. How the ANEMONE approach would be used in practice depends a lot on who the users are and their prior experience as well as their fields of expertise. Another related challenge is *how* the ANEMONE approach should be tested. One suggestion is to use Blandford and Green’s own framework, in particular the various criteria presented in the second phase [94], e.g., scope, validity, reliability, learnability, usability, and insights derived. Likewise, Nilsson [97] addresses structure, effectiveness, construct validity, and reliability. In addition, Halverson [119] provides the following four attributes: descriptive power, rhetorical power, inferential power, and application power. Descriptive power refers to the fact that a methodology should offer a conceptual framework that helps us make sense of and describe a messy world, including describing a work setting and evaluating the implementation of technology in the particular setting. Rhetorical power means that methodology should guide us in structuring and being able to talk about the world by denoting important aspects of the conceptual space and how it fits to the context. Inferential power is about the need for a theory that makes us make inferences, without battling in arguments and discussions about whether a theory is true or only falsifiable. Finally, application power refers to how well we could apply the methodology in practice for pragmatic reasons [119]. One may ask whether ANEMONE should be tested for all these criteria or a selection of some of them. Besides the criteria that could be used, it would be beneficial that other colleagues from various fields of expertise carry out the testing in practice, beyond conducting the testing phase by ourselves. Furthermore, the ANEMONE approach should be tested on various kinds of robots, in various kinds of settings, and with different end-user groups to provide relevant input on the applicability, construct validity, and structure of ANEMONE. However, like all data collection as well as testing, access to workplaces like manufacturing plants can be restricted due to productivity and commercial and safety issues, as well as the overall challenge of collecting relevant data in more fuzzy environments. Potential scenarios in which it could be tricky to use ANEMONE could be, i.e., when several persons are working together, the actions or activities are distributed over a larger physical area, the tasks are nested into other tasks/operations/activities, the tasks/actions/activities span several people and/or are distributed in time, and when the investigator has limited knowledge about the work carried out.

### 5.3. Future Work

**Figure 7:**
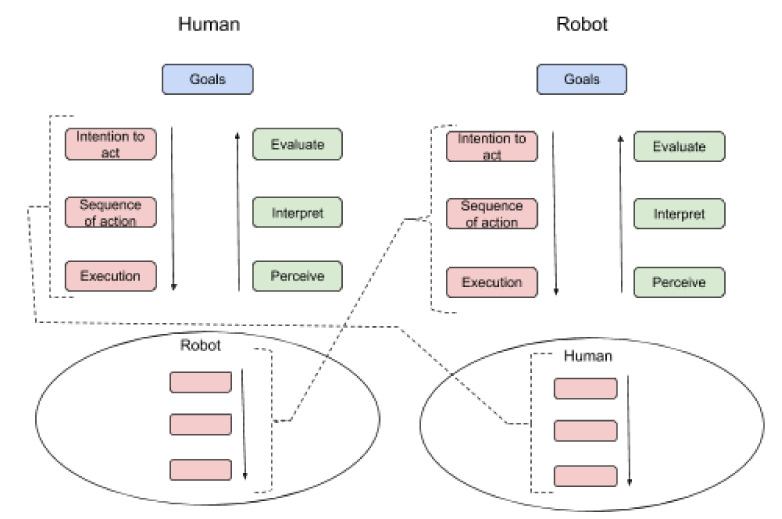
Expanding the seven stages of action model to mutual action and intention recognition between a human and a robot simultaneously.

The focus of the methodological approach is UX and is derived from the area of HCI. However, there are differences between interacting with a computer and a robot, not least that a robot, in the form of an autonomous intelligent system [61], moves in shared physical and social spaces. This makes it important not only to consider that a human can recognize the actions and intentions of a robot, but also the other way around, e.g., the robot can “perceive”, “understand”, and “predict” the actions and intentions of humans, although the robot itself lacks intrinsic intentionality. The issue of robot intentionality is beyond the scope of the proposed methodological approach, in which we rely on Dennett’s intentional stance [58] (see Section 1.1). Accordingly, there is a possibility to explore an expansion of the modified seven stages of action model that reflects the *duality* of the interaction, where both the human and the robot are active and physical agents (Figure 7). In this expanded model, the human and the robot in parallel (or simultaneously) carry out their actions, where the other agent is part of the context that is perceived, interpreted, and evaluated. In other words, while the robot is executing (the down-going path in the figure) the human evaluates (the up-going path in the figure) the robot as part of the context, and when the human executes the robot should ‘evaluate’ the human as a part of the context. This way of viewing the mutual need of action and intention recognition between humans and robots can form a basis for enlarging the scope of the current methodological approach of ANEMONE to also embrace the robot’s perspective.

The proposed UX evaluation approach, ANEMONE, is grounded in a well-established theory, methodology, and model, i.e., activity theory, UX evaluation methodology, and the seven stages of action, that separately have been thoroughly researched and validated in the area of HCI. They have previously not been put together in the field of human-robot interaction with the focus of UX-centered recognition of robots’ actions and intentions. The characteristics of the ANEMONE have been incrementally developed and validated in a series of workshops (see Section 2). Nonetheless, as a whole the approach has not yet been fully tested and validated in a human-robot evaluation setting in practice, which, hence, is the next step. This is currently work in progress and will result in another paper. Testing ANEMONE in practice does not only provide confirmation of validity, but also more details and nuances that can constitute a basis for developing the approach into an even more structured methodology with guidelines and concrete recommendations on how to use it for special purposes and aims.

Additional future work involves making a shorter version of ANEMONE, in the form of a more ‘quick and dirty’ empirical UX evaluation approach inspired by the activity checklist [104], as well as making a separate analytical UX evaluation approach. To conclude, we hope that ANEMONE provides a step forward to enable robots to be omnipresent and situated within our society as Stephanidis et al. [1] envision; for robots to become situated and embedded in our daily lives, they have to be suited for our purposes, fulfilling our emotional and personal needs, and be perceived and experienced positively by us.

Finally, we look forward to seeing and conducting more AT-influenced research on robot-mediated interaction on all levels of activity, from a UX perspective on action and intention recognition. The UX perspective is constantly putting the human user in the center in the evolving technological environments that humans are situated within, which are hybrid worlds that are envisioned to be inhabited by humans and various kinds of robots. Indeed, various efforts are needed in order to have “a deeply human(e) perspective on intelligent technologies” [1] (p. 1259) [24], and currently, there is a long journey ahead of us. We hope our work with ANEMONE will contribute to tackling this challenge, because the over-reaching goal of the UX field is to contribute to the quality of human life in these evolving hybrid worlds.

## Figures and Tables

**Table 1:** An overview of the phase-by-phase description of the procedure for action and intention recognition in human robot interaction (ANEMONE).

***Phase 1: Preparation***
1.1 Identification of case: identifying the area of interest and defining the context
1.2 Specify UX and evaluation goals
***Phase 2: Selection of UX evaluation type***
2.1 Evaluation scope
2.2. Characterization of analytical and empirical UX evaluation
***Phase 3: Plan and conduct the UX evaluation***
3.1 Analytical UX evaluation of action and intention recognition
3.2 Empirical UX evaluation of action and intention recognition
***Phase 4: Analysis of collected data and identifying UX problems***
4.1 Strategies for analysis of the collected data and identifying problems
4.2 Analysis and problem identification of action and intention recognition
***Phase 5: Organizing the identified UX problems in scope and severity***

**Table 2:** Focal points to consider during the analysis of the human-robot interaction situated within the context of interest to identify UX problems and tentatively gain a better understanding of *why* they occurred, which here are portrayed in the context of human-robot interaction in assembly (adapted from [104,117,121,122]).

Step	Focal Point(s)	Examples
1	Activity	The improvement of positive UX in human-robot interaction at the manufacturing floor where, e.g., the action and intention recognition between operator and robot has to be experienced as ‘smooth’ and ‘natural’ in order to be perceived trustworthy and safe for the operators during assembly of a part in an engine.
2	Objective	The motives that drive the operators to use available tools to convert the object of activity into valuable outcomes, e.g., positive UX, better product quality, and higher product quantity.
3	Actors (subjects)	Skilled manual assembly operators with no prior encounter of human-robot interaction in assembly.
4	Mediating artifact and tools	The collaborative robot itself is the main mediating artifact. Other relevant tools necessary to perform the assembly tasks (e.g., screw-drivers, tablets with instructions, components to be assembled) as well as previous knowledge and skills of the operators.
5	Rules	The implicit and explicit rules, norms, and procedures are relevant in the case at hand, e.g., safety regulations and other work-related rules, norms, and practices that regulate the use of collaborative robots on the manufacturing floor. The assembly has to be conducted at a fast pace (high saturation), with a low error rate, and with high production quality. Safety instructions have to be followed when working with the robot. Job rotation routines between different work stations.
6	Division of labor	The ways the responsibilities of value creation are distributed and divided among several actors (subjects) who participate in the community. What is the division of labor between the mediating artifact (i.e., robot) and the assembly worker in their collaboration? What division of labor is relevant among the persons involved in this case? The characteristics of task allocation and levels of collaboration between the operator and robot in the distribution of assembling at the work station, which can be scaled up to other operator and robot teams at additional work stations on the same line, and other personnel involved (line manager, plant manager etc.).
7	Community	The wide array of other relevant persons in this case that are involved in the activity, such as other operators and robot teams at additional work stations on the same line, and other personnel involved such as line managers, maintenance personnel, and line planners.
8	Outcomes	What is the expected outcome of the activity? A mounted part on a car engine which could be scaled up to the production of high quality products, e.g., car engines in high volumes. This production is performed with respect to, on the one hand, positive UX of the quality of human-robot interaction, job satisfaction and good working environment, well-being at work and, ideally, on the other hand, environmental, social, and economic sustainability of manufacturing systems.

**Table 3:** Guiding questions to raise during the analysis based on the interrelations within the activity system during the analysis (adapted from [104,121,122]).

Components of the Activity System	Questions to Ask During the Analysis
Actors (subjects) and mediating artifacts/tools	What mediating artifact(s) and tools does the user handle in order to achieve his/her objective(s) and how?
Tools and objects	What is the access to the mediating artifact and tools necessary to perform the activity? How is the spatial and temporal layout of the current workstation and the components to be assembled with a collaborative robot at the assembly line?
Mediating artifact/tools and community	How do the mediating artifact and other tools that are used affect the way the community achieves its objectives?
Rules and actors (subjects)	What rules, norms and regulations affect the way the activity is carried out and how? Do any potential conflicts exist between rules, norms, and practices and the user’s goals?
Rules and community	What kind of rules affect the way the community satisfies their objective and goals? It could be human factors and environmental issues like working hours allotted to work during nightshifts and energy consumption
Actors (subjects), mediating artifacts/tools and activity (hierarchal structure of activity)	How and when are the mediating artifact and tools handled by the users in order to achieve the activity? How are various mediating artifacts and tools integrated and used? Do any workarounds exist?
